# Chaotic Direct Ink Writing (ChDIW) of Hybrid Hydrogels: Implication for Fabrication of Micro‐ordered Multifunctional Cryogels

**DOI:** 10.1002/smtd.202500349

**Published:** 2025-03-13

**Authors:** Shakiba Samsami, Zahra Monsef Khoshhesab, Juan Felipe Yee‐de León, Diego Alonso Quevedo Moreno, Mario Moisés Alvarez, Grissel Trujillo‐de Santiago, Kam C. Tam, Milad Kamkar

**Affiliations:** ^1^ Department of Chemical Engineering, Waterloo Institute for Nanotechnology University of Waterloo Waterloo Ontario N2L 3G1 Canada; ^2^ Departamento de Ingeniería Mecatrónica Tecnologico de Monterrey Monterrey NL 64849 Mexico; ^3^ Department of Mechanical Engineering Massachusetts Institute of Technology Cambridge MA 02139 USA

**Keywords:** chaotic flows, direct ink writing, electromagnetic interference shielding, multilayered cryogel, multimaterial printing

## Abstract

The modern era demands multifunctional materials to support advanced technologies and tackle complex environmental issues caused by these innovations. Consequently, material hybridization has garnered significant attention as a strategy to design materials with prescribed multifunctional properties. Drawing inspiration from nature, a multi‐scale material design approach is proposed to produce 3D‐shaped hybrid materials by combining chaotic flows with direct ink writing (ChDIW). This approach enables the formation of predictable multilayered filaments with tunable microscale internal architectures using just a single printhead. By assigning different nanomaterials to each layer, 3D‐printed hydrogels and cryogels with diverse functionalities, such as electrical conductivity and magnetism are successfully produced. Furthermore, control over the microscale pore morphology within each cryogel filament is achieved, resulting in a side‐by‐side dual‐pore network sharing a large interfacial area. The ChDIW is compatible with different types of hydrogels as long as the rheological features of the printing materials are well‐regulated. To showcase the potential of these multilayered cryogels, their electromagnetic interference shielding performance is evaluated, and they reveal an absorption‐dominant mechanism with an excellent absorption coefficient of 0.71. This work opens new avenues in soft matter and cryogel engineering, demonstrating how simplicity can generate complexity.

## Graphical abstract

Multimaterial 3D printing is being used for the development of highly efficient and multifunctional materials capable of meeting both complicated technological needs and addressing the extreme environmental challenges posed by modern life. This study explores the combination of the microdesign dictated by chaotic flows with the macrodesign provided by the direct ink writing technique to achieve multifunctional 3D‐printed hydrogels and cryogels featuring novel morphologies.

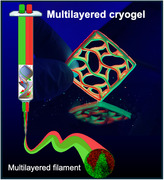

## 1 Introduction

Multimaterial 3D printing has opened up new frontiers in the design and fabrication of advanced structures. The performance and functionality of these structures are largely determined by the depth of the synergy between the materials involved. While single‐material 3D printing limits the range of reachable properties and requires a trade‐off between desired characteristics, multi‐material 3D printing efficiently addresses these limitations by integrating diverse materials with distinct properties into a single structure. The concept of extrusion‐based multi‐material 3D printing has been demonstrated by several studies so far.^[^ ^1^, ^2^ ^]^ Considering these different methods, single‐nozzle co‐printing approaches exhibit many advantages compared to multi‐nozzle co‐printing or sequential printing techniques. While sequential printing methods may have limited printing resolution and slow printing speed,^[^ ^3^, ^4^ ^]^ multi‐nozzle co‐axial printing methods involve complicated systems with a high cost of maintenance and a lack of printing resolution at the microscale.^[^ ^5^, ^6^ ^]^

Single‐nozzle co‐printing methods can achieve high resolution through either optimized nozzle or cartridge designs at a lower cost. In terms of nozzle design, besides 3D core–shell structures,^[^ ^7^ ^]^ Larson et al. developed a novel continuously rotating multi‐material nozzle to fabricate helical filaments, engineering artificial muscles.^[^ ^8^ ^]^ Notably, this platform allowed for individually addressable conductive helical channels embedded within an elastomer matrix. Another tactic to co‐print multiple inks via one printhead is designing the cartridge instead of the nozzle. For instance, Kang et al. fabricated such a system by developing a pre‐set extrusion bio‐printing method including a cartridge that could preserve multiple materials in a pre‐defined shape in a syringe‐based printhead.^[^ ^9^ ^]^ They utilized this printing platform to fabricate heterogeneous artificial tissue‐like structures.

Nonetheless, single‐nozzle methods still lack flexibility for selective nanomaterial deposition in a simple and cost‐effective manner. Another research gap is the limited focus on creating multilayered structures, which are crucial for many applications such as electromagnetic interference (EMI) shielding, sensors, adsorption, energy storage, and biomedical applications. Altogether, simple and versatile one‐step multi‐material 3D printing strategies that efficiently enhance the accuracy of structural control, especially multilayered structures, without adding complexity still call for modern engineering innovations.

Chaotic flow, considered as one of the most efficient mixing approaches in the laminar regime, can be an appealing candidate for enhancing the micro‐structural control of multi‐material 3D printing.^[^ ^10^ ^]^ Chaos colloquially refers to randomness, however, in ironic contrast, physical chaos produces highly ordered patterns controlled by deterministic laws.^[^ ^11^ ^]^ It is one of nature's key inspiring strategies that facilitates the creation of multilayered structures. Inspired by nature, chaotic flow is a novel approach to meticulously develop multilayered hybrid filaments with highly tunable internal architecture that can be mathematically modeled and predicted. In 2018, Trujillo‐de Santiago et al. proposed a novel and cost‐effective platform to develop predictable multilayered constructs with tunable fine internal multilayered micro‐architecture.^[^ ^12^ ^]^ In this approach, the chaotic mixing via the Kenics static mixer (KSM), which has wide applications in various industries, is not used until homogeneity is achieved. However, the extent of chaotic mixing is dictated by the number of mixing elements, determining the resolution of the layers.^[^ ^13^ ^]^ Apart from the convenience and efficiency of this method, one crucial advantage of using chaotic flows for multi‐material 3D printing is achieving and adjusting large interface areas between multiple involved materials at a very high speed and resolution.^[^ ^14^, ^15^, ^16^ ^]^ This merit benefits any applications in which interfaces play an important role,^[^ ^15^ ^]^ such as living tissues,^[^ ^14^, ^17^ ^]^ hydrogel carriers,^[^ ^18^, ^19^ ^]^ and bacterial communities.^[^ ^20^ ^]^ However, despite many remarkable achievements in employing chaotic flows to precisely fabricate hybrid microstructures, the control over their macroscale design has been relatively overlooked. Thus far, few advantages have been achieved from combining the chaotic flows with other technologies to further attain control over upper scales. There is only one report on utilizing it in a hybrid system of chaotic electrospinning.^[^ ^21^ ^]^ More importantly, the potential of chaotic printing for constructing multilayered aerogels or cryogels has not yet been examined.

Herein, for the first time, we coupled the micro‐design dictated by the chaotic flows with the macro‐design provided by the direct ink writing (DIW) technique to achieve highly precise multifunctional 3D‐printed hydrogels and cryogels. This hybrid system greatly simplifies the multi‐material 3D printing procedure and allows us to co‐extrude two inks continuously with only one printhead. For the inks, hydrogels are optimized to produce multilayered viscoelastic filaments. Cellulose nanocrystal (CNC)‐based hydrogels are selected as they are sustainable and meet the required criteria for DIW. To endow the multilayered 3D printed hydrogels and their resultant cryogels with multifunctionality and independent tunability, two different inks with distinctive properties are assessed: 1) electrically conductive and 2) magnetic CNC‐based hydrogels. To the best of our knowledge, this is the first report examining the chaotic DIW (ChDIW) approach in preparing hybrid hydrogels and cryogels. The produced cryogels are composed of alternating microlayers with different porosities sharing large surface areas. By leveraging the ability of ChDIW to fabricate multilayered structures with controlled properties, this study expands the utility of this technology into entirely new fields. For instance, the development of chaotically printed cryogels with non‐interfering magnetic and conductive layers represents an innovation in the field of electromagnetic interference (EMI) shielding. The alternating magnetic/conductive layers within each filament exceed existing shielding materials by offering an absorption‐dominant mechanism, addressing a prevailing challenge in the EMI shielding field.

## 2 Results and Discussion

### 2.1 Concept and Preliminary Assessments

The fabrication of multilayered cryogels via the proposed hybrid chaotic direct ink writing (ChDIW) approach requires multi‐scale material design as follows: 1) designing 3D printable inks with fine‐tuned nano‐scale building blocks, 2) creating multilayered filaments with controlled microscale non‐interfering layers via chaotic mixing process using a kenics static mixer (KSM), 3) 3D shaping the generated multilayered filaments on the macroscale via the DIW technique, and finally 4) transforming the chaotically 3D‐printed hydrogels into multifunctional cryogels via a one‐step freeze drying approach while preserving the architecture at the mentioned scales. By using the ChDIW‐processed hydrogels, it is now possible to produce cryogel filaments with distinct layers that incorporate different engineered nanomaterials and exhibit various microscale porosities.

Each of the proposed steps necessitates precise materials engineering at the respective scale. Initially, to form and preserve the meticulous microscale design of the internal layers produced in the chaotic mixing step, three critical factors should be adjusted: the number of mixing elements within the KSM printhead, the chemical/physical compatibility, and the laminar flow of two different inks. Next, the DIW of the generated multilayered filaments with high quality obliges close rheological properties of the inks to guarantee their shape fidelity during extrusion and after deposition. For instance, the inks should exhibit a liquid‐like behavior for a smooth flow in the needle under high shear stress and recover their elastic nature immediately after deposition on the bed to prevent lateral spreading. Also, adjusting the needle size attached to the KSM printhead is an important step to preserve the internal layers after passing through the needle. Finally, to stabilize the generated internal microlayers in the cryogel form, the chemical potential of the materials to form interactions attaching them to each other at the interface plays the main role. Hence, several criteria must be met to produce multilayer 3D‐structured cryogels using the ChDIW method all of which are addressed systematically and discussed in the following sections.

**Figure 1:**
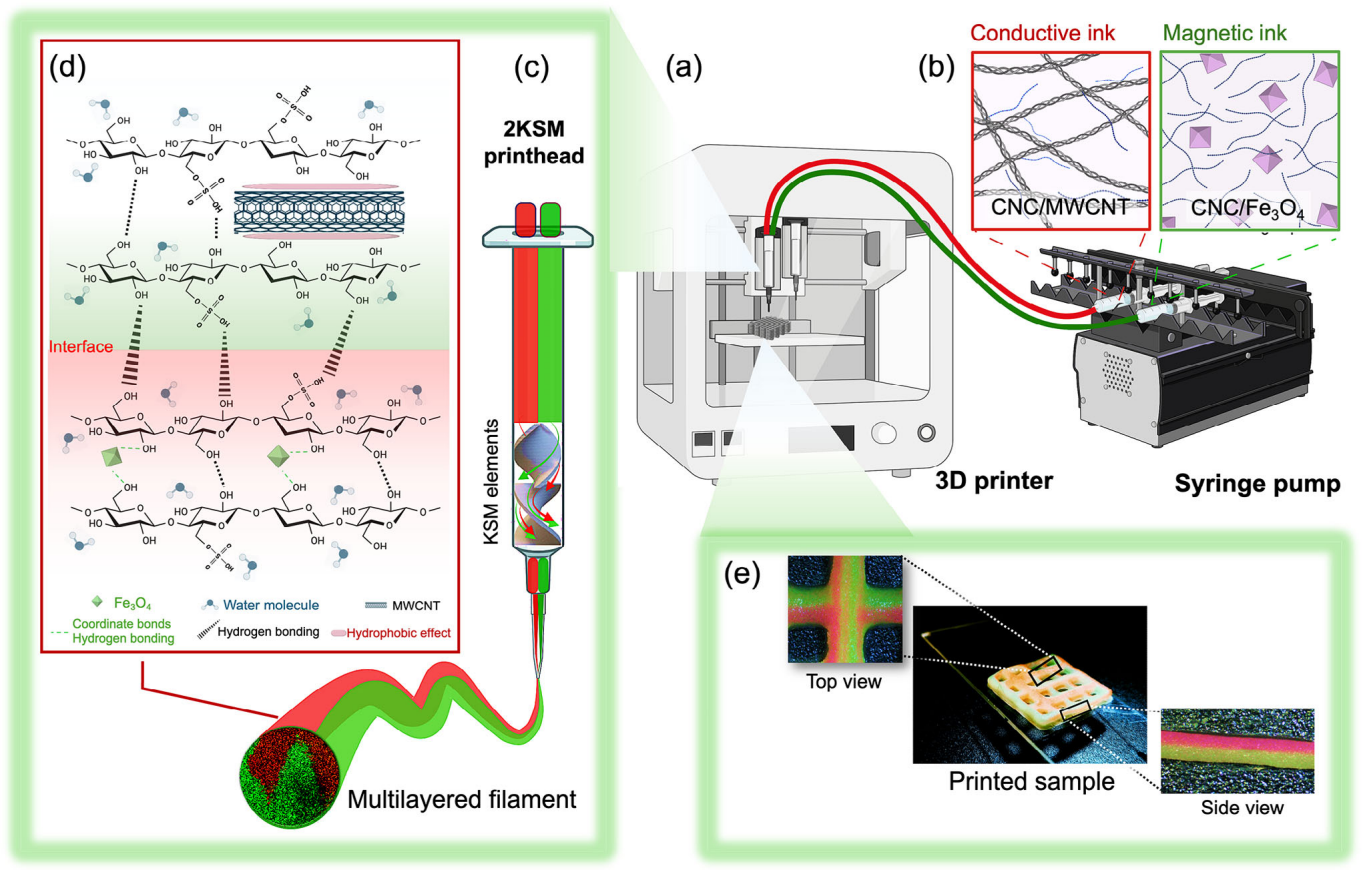
a) Illustration of the ChDIW system consisting of a 3D printer and a syringe pump for delivering b) two different functional hydrogels into the 2KSM printhead. c) Schematic representation of the 2KSM printhead with two inlets on the lid and two KSM elements implemented for the co‐extrusion of magnetic and conductive inks, forming a filament with inner multiple layers. d) Proposed interactions between two different functional inks at their interface within each multilayered filament produced by the chaotic advection. Created in BioRender. Samsami, S. (2025) BioRender.com/ a63f021. e) Photographs of the chaotically printed sample by the 2KSM printhead fed up with pure CNC 15 wt% hydrogels dyed with red and green fluorescent particles for visualizing the multilayered filaments within the final 3D structure.

To achieve the desired goals, other than engineering the inks, the ChDIW system was designed, which was composed of a syringe pump for delivering inks into the KSM printhead and then co‐extruding through the needle and a 3D printer to ultimately shape them (**Figure** **1**a). This hybrid system included two main stages: chaotic mixing and DIW. As illustrated in Figure 1c, the chaotic mixing occurred through the static mixer containing various mixing elements within the KSM printhead that allowed the continuous co‐extrusion of the flowing hydrogels. The mixing elements are plates that have been designed with a 180° helical twist and are serially placed inside a cylindrical tube. Each KSM element in this serial is rotated 90° with respect to the previous element. As the hydrogels pass through the KSM printhead, the helix‐shaped mixing elements bifurcate and double the number of the existing layers in the previous mixing element.^[^ ^10^ ^]^ As a result, a layered structure is formed by iterative actions of stretching and folding, increasing the number of generated layers exponentially.^[^ ^10^ ^]^ This self‐similar nature of chaotic flows was also evident in simulation results achieved by computational fluid dynamics (CFD) strategies that can be found in our previously reported works.^[^ ^20^ ^]^

**Figure 2:**
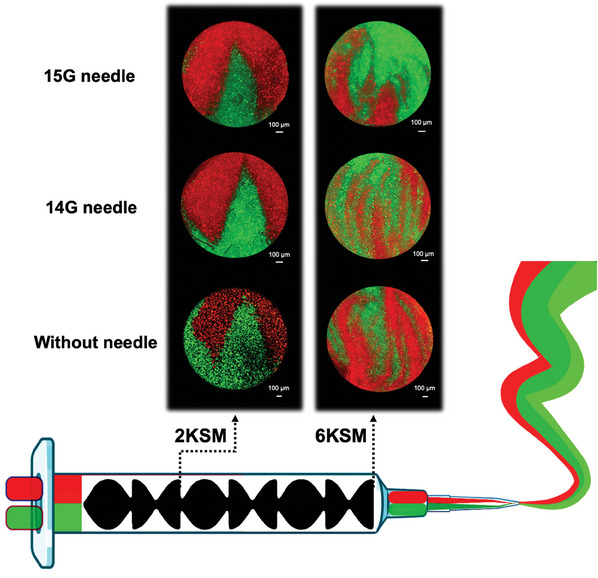
Cross‐sectional views of the multilayered filaments containing CNC 15 wt% hydrogels produced by 2KSM and 6KSM printheads without and with two different needle sizes (flow rate = 1.5 mL min^−1^).

To address the first prerequisite of the ChDIW process, evaluating the capability of the KSM printhead to co‐extrude and produce multilayered filaments of “paste‐like” hydrogels with precise micro‐order morphologies, 15 wt% cellulose nanocrystal (CNC) hydrogels with the same rheological properties were used. To distinguish the layers and showcase the successful ChDIW process, the inks were stained with two different fluorescent colors, i.e., red and green. The high viscosity of this hydrogel also ensured the laminar flow (see Figure S1, Supporting Information). Herein, two KSM printheads, i.e., 2KSM and 6KSM, were utilized to assess the effect of different numbers of mixing elements. Also, to evaluate the stability of the internal structure of the filaments under different shear stresses, different needle sizes were installed on the KSM printheads. As depicted in **Figure** **2** (filaments’ cross sections), the 2KSM printhead without any needle successfully constructed filaments featuring predicted four (= 2^2^) microlayers of the CNC hydrogels within the filaments, whereas the 6KSM printhead failed to produce the expected 64 (= 2^6^) internal microlayers and approached a mixed state as a result of chaotic mixing. When both printheads were equipped with needles, only the 2KSM printhead produced the expected cross‐sectional structure in terms of the number and design of the microlayers. This could be generally attributed to the higher shear stress induced by the needle and wall irregularities induced by the joint of the chaotic mixer and the needle that caused flow disturbances, altering the inner multilayered structure. Also, the smaller microlayers created by the 6KSM printhead could not withstand this increased shear stress while the larger microlayers by the 2KSM printhead resisted it, conserving the organized microlayers.

Remarkably, changing the needle size did not make any substantial differences in the internal structure of the filaments made by the 2KSM printhead, proving its promising stability at different filament sizes. The chemical compatibility and laminar flow of these hydrogels were responsible for producing the fine interfaces between them after passing through the mixing elements. Additionally, adjusting the number of mixing elements was vital for conserving the predicted interfaces after passing through the needle. Henceforth, the 2KSM printhead was selected for the DIW process as the next step of the ChDIW method to shape the produced multilayered filaments at the macroscale, patterning multilayered hydrogels and cryogels.

**Figure 3:**
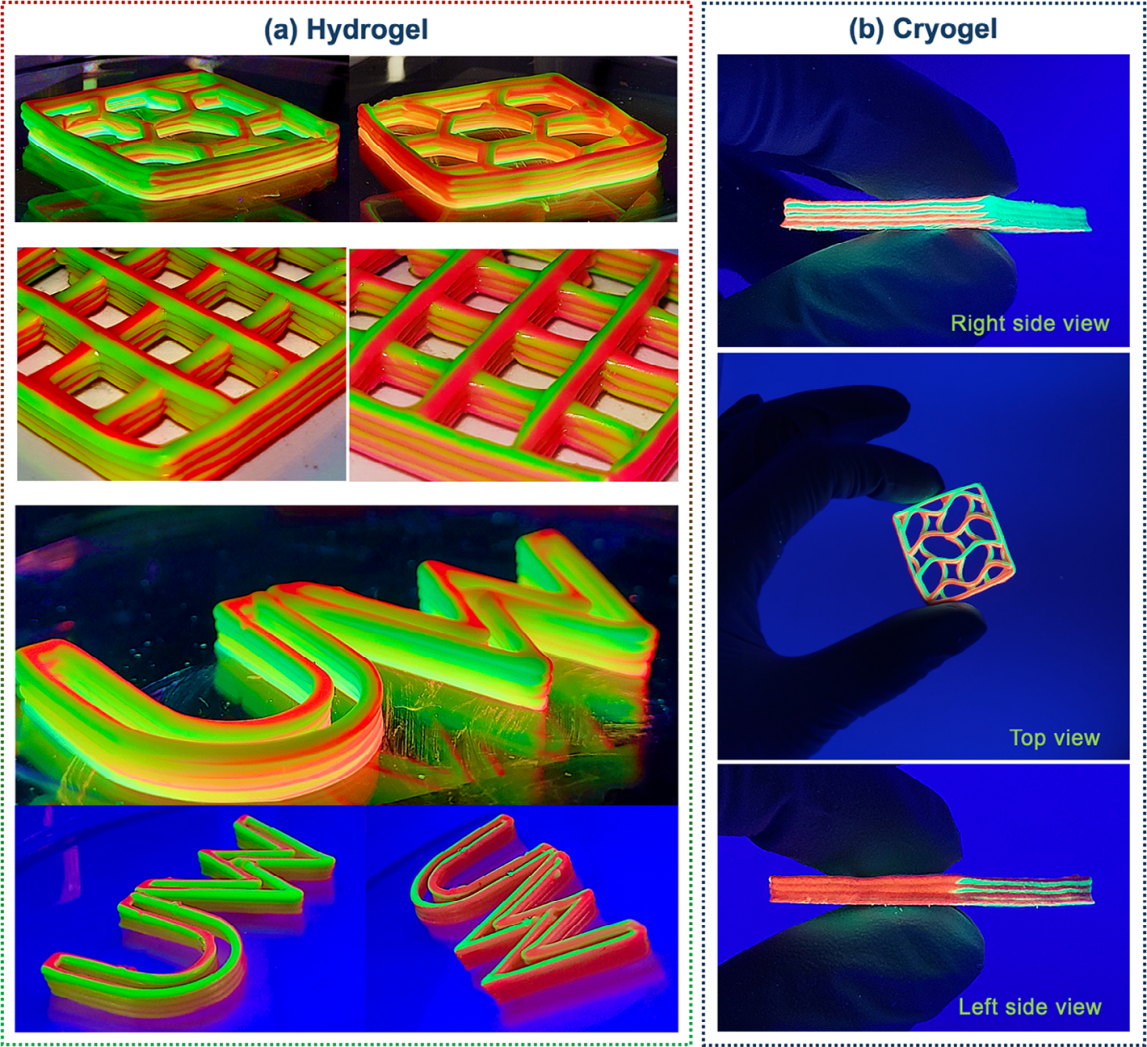
The micro‐ and macro‐design quality of the ChDIW technique for producing a) hydrogels and b) cryogels with various designs and shapes. These images indicate the excellent layer resolution, structural integrity before and after drying, and different characteristics when observed from different sides (printing parameters: 2KSM printhead, 18G needle, flow rate = 0.8 mL min^−1^, printed object dimensions = length and width 3 cm, and height 3 mm).

To further design the multilayered filaments at the macroscale, the ChDIW was utilized to chaotically print the paste‐like CNC hydrogels with the optimized 2KSM printhead. The top and side views of each filament within the final 3D structure are displayed in Figure 1e. Extensively, **Figure** **3**a and Video S1 (Supporting Information) showcase the high‐quality micro‐ and macro design of the ChDIW hybrid technique, featuring various designs and shapes that demonstrate precise layer resolution and structural integrity. The variations in geometry highlight the printing method's adaptability for diverse applications. Figure 3a indicates that the multilayered 3D‐printed structure displayed different characteristics when observed from different sides. When viewed from one side, the object predominantly revealed one ink (in green color), while viewing from the other side, mainly displayed the other ink (in red color). This also confirms the accuracy of the KSM printhead in producing a reliably meticulous internal structure, as it requires each filament to consistently maintain a precise interface between the two different inks. This phenomenon is reminiscent of the Janus effect, where materials exhibit distinct properties on opposite sides. However, unlike typical Janus materials, the structure produced by the ChDIW technique achieved a homogeneous distribution of different material properties throughout the final product, enabled by its unique micro‐design capability. This approach enhances the overall uniformity while preserving the distinct visual and functional variations, making it a compelling and innovative example of multi‐material 3D printing.

Figure 3b confirms that the shaped internal interfaces could be preserved not only once deposited, thanks to the inks’ compatible viscoelastic characteristics, but also after drying due to the inks’ compatible chemical properties. This achievement was noteworthy since it demonstrated that the hybrid ChDIW process has appreciable control over both micro‐ and macroscale design of the final structure that can be stabilized after drying. Moreover, this structure has the potential to be adjusted at the nanoscale to serve multifunctionality due to offering the non‐interfering alternating inner layers. This could be achieved by the incorporation of distinct functional nanomaterials in each layer, which will be discussed and showcased in the next section.

Altogether, the one‐step ChDIW technique offers many advantages due to the developed synergy among the chaotic flows and DIW process. The high viscosity of the paste‐like hydrogels (as a requirement of the DIW method) safeguards the upkeeping of the laminar flow in the chaotic mixing step. Concurrently, chaotic mixing facilitates the construction of the multifunctional hybrid 3D‐printed structures with adjustable microscale design. Remarkably, these benefits can be attained with just one printhead, leading to reduced energy consumption and lower production costs and time.

The straightforward tunability on multi‐scales and energy‐ and cost‐efficiency provided by the ChDIW technique motivated us to delve deeper into its prospective merits to fabricate hybrid multifunctional 3D‐printed hydrogels and cryogels. To achieve this, different functional CNC‐based inks were formulated. Each CNC hydrogel was endowed with different functions and properties by controlling their nano‐scale design: i) electrical conductivity by adding multi‐walled carbon nanotubes (MWCNTs) and ii) magnetic properties by adding iron oxide nanoparticles (Fe_3_O_4_). Primarily, magnetic and conductive CNC‐based hydrogels had to be optimized separately considering their rheological behavior for high‐fidelity DIW printing. The following sections describe how the inks were systematically optimized, characterized, and formulated for the ChDIW process. The prepared conductive and magnetic hydrogels were labeled as (C4, C6, and C8), and (M1, M2, and M3), respectively. The details of the gel formulations are given in Table S1 (Supporting Information).

### 2.2 Multifunctional Structures by the ChDIW Technique: Nanoscale Design

Two functional CNC‐based hydrogels were independently optimized and co‐extruded through the 2KSM printhead. Separately, Fe_3_O_4_ nanoparticles and MWCNTs were dispersed within a CNC hydrogel network by the coordination bonds and hydrophobic effects, respectively (Figures 1b,d). Details of the ink preparation can be found in the Supporting Information. In summary, Fe_3_O_4_ nanoparticles were dispersed in the CNC hydrogels by homogenization while a sonication‐assisted technique was used for the fabrication of 3D printable MWCNTs/CNC hydrogels. The flow behavior of all prepared conductive and magnetic inks exhibited shear‐thinning behavior, confirming their smooth extrudability through the nozzle under sufficient shear stress (Figures S2a,d, Supporting Information). Also, the viscoelastic properties of the gels as the central features of any extrusion‐based process were further examined, ensuring their shape fidelity. Figures S2b–f (Supporting Information) depict the loss (*G*′′) and storage (*G*′) modulus of the gels versus strain amplitude and angular frequency. The dynamic moduli of all the magnetic and conductive suspensions displayed a frequency‐independent trend, confirming the dominance of a gel‐like behavior.

Although the shear‐thinning behavior and gel‐like nature of the suspensions were promising factors for the printability; to select the best printable gel, all the gels were printed under the same printing conditions. As shown in Figure S3 (Supporting Information), among the conductive gels, only the C6 hydrogel showed excellent printability, so it was selected as the optimal conductive ink. Also, among the magnetic gels, the M2 gel provided higher shape accuracy and printing quality, so it was considered as the optimal magnetic ink. Videos S2 and S3 (Supporting Information) show the excellent printability of C6 and M2 inks, respectively. Additionally, Figures 3a_1_,b_1_ represent the stability and shape accuracy of 3D samples with different patterns printed by the C6 and M2 inks, respectively. Hereafter, the next assessments of the ChDIW process were performed on the C6 and M2 gels as the optimal conductive and magnetic inks, respectively. A comprehensive discussion on optimizing the prepared gels can be found in the Supporting Information.

**Figure 4:**
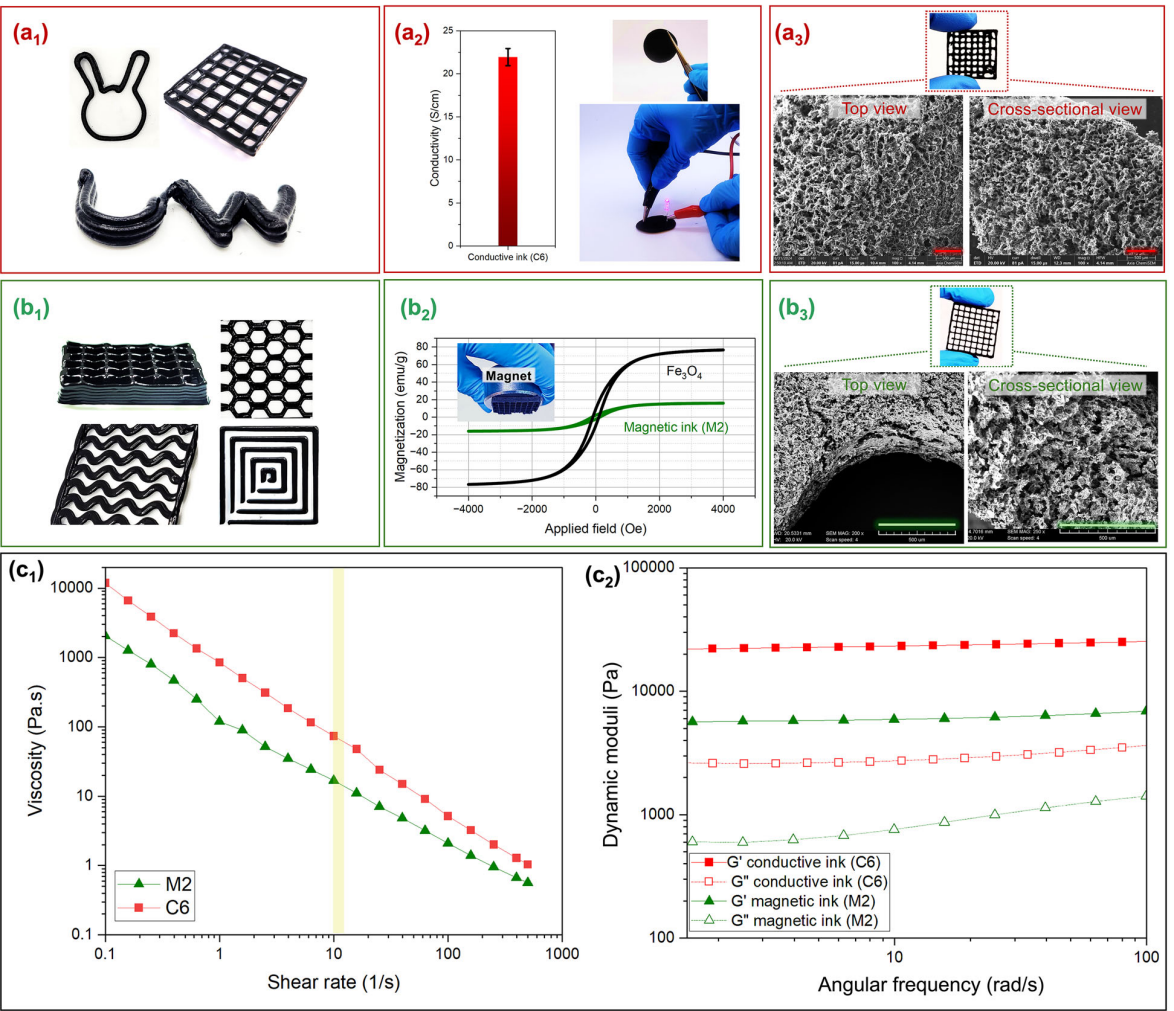
Characterization of the optimized conductive and magnetic inks. a_1_–a_3_) Conductive ink characterizations: a_1_) printability for various patterns, a_2_) electrical conductivity, and a_3_) SEM images of the optimized conductive C6 ink. b_1_–b_3_) Magnetic ink characterizations: b_1_) printing quality for different infill patterns, b_2_) magnetic properties, and b_3_) SEM images of the optimized magnetic M2 ink. All the scale bars on the SEM images represent 500 µm. c_1_–c_2_) C6 and M2 inks rheological characterizations: c_1_) viscosity of both inks versus shear rate, and c_2_) storage and loss moduli of both inks versus angular frequency.

After optimizing the gels considering their rheological behavior and DIW printability, the conductivity and magnetic properties of the final formulations were evaluated. To assess the electrical conductivity of the C6 ink, a four‐point probe measurement was conducted. **Figure** **4**a_2_ indicates that the electrical conductivity of a film made from C6 ink reached 22 ± 1 S cm^−1^, lighting up the LED diode. The uniform conductivity of different spots on the sample also confirmed the production of a homogenous dispersion of the MWCNTs all over the CNC/MWCNT gel network. The homogenous network and porous structure of the cryogels made from C6 hydrogel were also verified by the SEM images from the side and cross‐sectional views (see Figure 4a_3_).

The magnetic characteristic of the optimal M2 ink was evaluated by applying a uniform magnetic field to the sample oscillating perpendicularly to this field during the vibrating sample magnetometer (VSM) test,^[^ ^22^ ^]^ and the results are shown in Figure 4b_2_. The extremely low remanent magnetization suggested that the material could not retain any permanent magnetization after removing the external magnetic field. Also, very low coercivity indicated that the material did not require a significant amount of reverse field to reduce the magnetization to zero after being saturated. These two factors, occurring due to the quick fluctuation of the magnetic moments of the M2 sample, suggested a nearly superparamagnetic behavior, revealing single domains of the nanoparticles. Besides, the saturation magnetization of the M2 ink decreased significantly in comparison to that of pure Fe_3_O_4_ magnetic nanoparticles (16 vs 77 emu g^−1^) due to the non‐magnetic nature of CNCs. From the structural design viewpoint, Figure 4b_3_ represents the top and cross‐sectional views of the M2 magnetic cryogel. Figure S4 also shows the well dispersion of Fe_3_O_4_ nanoparticles all over the structure without any noticeable aggregation. Comparing Figures 4a_3_,b_3_, different microscale porous structures were observed, resulting from the incorporation of different nano‐scale building blocks in the hydrogels.

Up to this point, the conductive C6 and magnetic M2 inks were systematically optimized at the nanoscale, considering their DIW printability. For the next step, the potential of these inks to generate and maintain the internal microlayers within individual filaments via chaotic mixing was investigated.

### 2.3 Multifunctional Structures by the ChDIW Technique: Microscale Design

**Figure 5:**
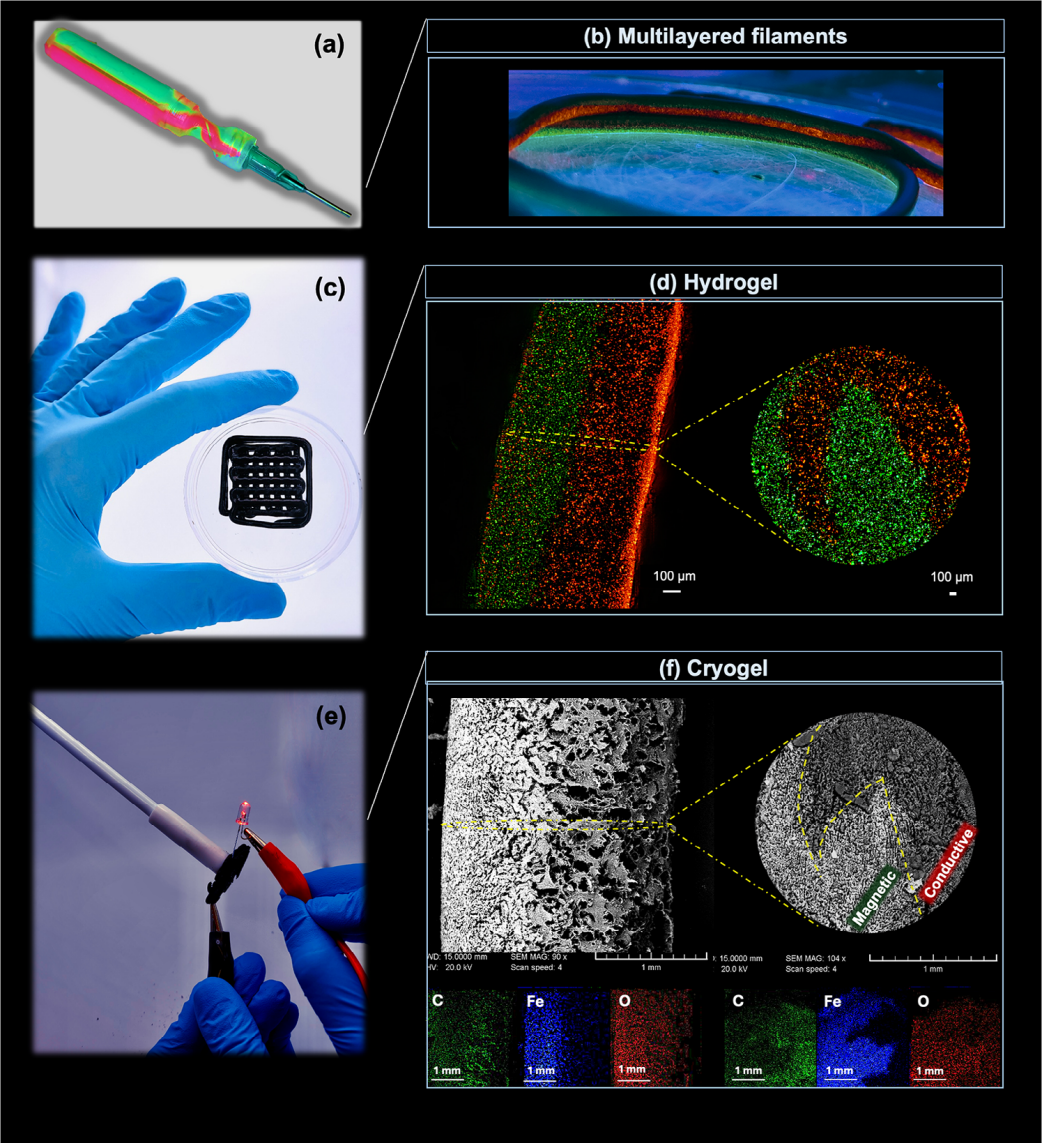
a) 2KSM printhead used in this study. The CNC‐based hydrogels were dyed with red and green fluorescent particles for visualizing the chaotic mixing process inside the printhead. b) Side view of two multilayered filaments next to each other under the UV light. Filaments contained C6 conductive and M2 magnetic inks dyed with red and green fluorescent particles, respectively. c) Chaotically direct ink written sample in the hydrogel form containing C6 and M2 inks. d) Fluorescence microscopic images of the side and cross‐sectional views of each filament within the chaotically printed sample, showing the successful creation of interfaces in the hydrogel form. e) Chaotically printed cryogel exhibiting both magnetic properties and electrical conductivity. f) Morphology (SEM) and elemental mapping (EDS) of the side and cross‐sectional views of each filament within the chaotically printed sample, verifying the preservation of the created interfaces in the cryogel form.

As discussed earlier, to fabricate multifunctional filaments with well‐ordered inner conductive and magnetic microlayers, controlling their chemical compatibility and laminar flow are the essential factors for co‐extrudability. The chemical compatibility was satisfied by employing water‐based gels for both inks, enabling the inks to flow stably side by side. The flow curves of the M2 and C6 inks also exhibited adequate viscosities of both inks to ensure their laminar flow at a shear rate of 11 s^−1^ during the ChDIW printing process (see Figure 4c_1_). The related calculations of shear rates and the very low Reynolds numbers of the two inks can be found in the Supporting Information. After addressing the first prerequisite, M2 and C6 inks yielded coherent multilayered filaments with the optimal 2KSM printhead, as depicted in **Figures** **5**a,b. Indeed, producing a multifunctional filament with an adjustable microscale arrangement using only one printhead is remarkable for the DIW process. This outstanding structure offered not only multifunctionality but also a large interface surface area between the inks that is readily customizable by the KSM printhead. Moving forward, these filaments were shaped by the DIW process via the hybrid ChDIW technique.

### 2.4 Multifunctional Structures by the ChDIW Technique: Macroscale Design

#### 2.4.1 Hydrogel

To successfully shape the formed multilayered filaments at the macroscale via the ChDIW technique, the 2KSM printhead was fed by the syringe pump loaded on a 3D printer (see Figure S5a,b, Supporting Information). The DIW process required close viscoelastic properties of the optimized inks to yield co‐printability and shape fidelity once deposited. As depicted in Figure 4c_2_, the oscillatory test results revealed comparable dynamic moduli of C6 and M2 inks over the entire frequency range, showing their promising co‐printability. Additionally, the deposited inks had to exhibit sufficiently high yield stress to resist the weight of the subsequent layers. To examine this quality, the maximum achievable height of the final multi‐material sample was found to be approximately 13 mm (see Supporting Information), safeguarding the self‐supported aspect of the chaotically printed sample with 5 mm height. Notably, the yield stress of the optimized C6 and M2 hydrogels was measured by conducting multiple creep tests by decreasing the applied stress from 600 to 150 Pa. According to Figure S6 (Supporting Information), the yield stress of C6 and M2 hydrogels was determined to be in the range of 150–200 and 250–300 Pa, respectively. At these specific ranges, no significant changes in the materials’ strain were observed and when the stress exceeded this level, the materials started flowing and the strain began to increase. Therefore, the rheological assessments suggested that the C6 and M2 inks had the potential to maintain their shapes side by side after ChDIW deposition.

As expected, Figures 5c and S5c (Supporting Information) show the excellent ChDIW‐printability of the optimized conductive and magnetic inks. The fluorescence microscopic images of the produced multilayered filament's side view within the 3D‐printed structure (Figure 5d) show the formation of a clearly defined interface between two hydrogels, confirming the adhesion of the inks to the interface induced by the chaotic advection. The alternating layers of the C6 and M2 existing in each filament were simply differentiable by the fluorescence microscopic images from the cross‐section of the filament. Based on these observations, it was revealed that for macro‐designing a chaotically printed structure by the proposed ChDIW technique, adjusting the rheological properties to formulate paste‐like hydrogels featuring high viscosity, dynamic modulus, and yield stress is of utmost importance.

#### 2.4.2 Cryogel

After taking steps to ensure that the micro‐ and macroscale designs were finely tuned for the ChDIW‐printed multilayered hydrogel, the capability of the materials to preserve their internal structure after drying was an important accomplishment and a milestone in this study. Cryogels offer large specific surface areas and porosity, low bulk density, and more structural stability compared to hydrogels.^[^ ^23^, ^24^ ^]^ In this study, the chaotically printed samples were simply freeze‐dried and transformed into lightweight magnetic/conductive cryogels (see Figure 5e). Their morphology was inspected by SEM analysis and is shown in Figure 5f. Fascinatingly, the chaotically printed cryogel exhibited flawless and distinctive boundaries between magnetic and conductive domains in both cross‐sectional and side views of the filaments. Dissimilar morphologies of the inks were clearly apparent across the interface. This observation confirmed that the inks could maintain their structures even after drying, preserving the well‐ordered and vast interface areas between them. More importantly, the SEM images showed how the hybrid ChDIW technique enabled the creation of interconnected pore networks of varying sizes within a filament. The dual‐pore network materials typically achieve a balance between rigidity and flexibility and are anticipated for multifunctional utilization, such as filters, absorbers, sensors, and biomedical applications.^[^ ^25^ ^]^ The chemical compatibility of the two inks played an important role in the production of the desired microstructure and morphology even after drying. Since water and cellulose were present on both sides of the interfaces, hydrogen bonds between two inks (see Figure 1d) functioned as a bridge to effectively connect and bond the domains together. This interaction between distinct hydrogels is a prerequisite to firmly adhere the adjacent microlayers together, ensuring the overall structural integrity before and after drying. If two inks were not chemically compatible or capable of forming sufficient interaction at the interface, the final multilayered structure would collapse and/or delaminate before or after drying. In addition, if two inks did not exhibit adequately close rheological properties, particularly yield stress, the final printed multilayered structure would deform or sag toward the ink with the lower yield stress, compromising the precision of the interfaces’ formation and retention.

To further inspect the presence of distinctive conductive and magnetic nanomaterials in each layer in the cryogel, EDS analysis was applied. As demonstrated in Figure 5f, the elemental mapping acquired by this analysis confirmed that the Fe_3_O_4_ nanoparticles were present in the defined layers allocated to the magnetic ink. The carbon and oxygen elements were present all over the sample since both inks were cellulose‐based hydrogels. However, the elemental mapping revealed variations in their intensities across the interface, reflecting differences in their localized concentrations. This could be attributed to the presence of more carbon elements in the conductive layers due to the existence of MWCNTs, compared to the magnetic layers. Analogously, the magnetic layers contained iron oxide nanoparticles as confirmed by the higher oxygen elements compared to the conductive layers.

In essence, these findings support the overall strategy of producing hybrid multilayered cryogel using the ChDIW method. The micro‐design was dictated by the chaotic mixing, essentially influenced by the chemical compatibility and laminar flow of both inks, as well as the number of KSM elements. On the other hand, the macro‐design was dictated by the DIW process and was directly adjustable by the close yield stress and viscoelastic properties of the involved inks. Subsequently, the preservation of the internal microlayers in the cryogels required materials with the capability of forming bonds between the two inks at the interface. According to the aforementioned criteria, the ChDIW system showed the potential to be one of the most versatile methods for producing hybrid multifunctional 3D printed soft materials and cryogels. The non‐interfering multilayered cryogels with dual‐pore networks lay a foundation for any application that demands multifunctionality.

### 2.5 A Potential Application for the Multifunctional Structures by the ChDIW Technique: Absorption‐Dominant EMI Shielding

To underscore the future potential of such 3D multilayered construct, magnetic/conductive cryogels prepared via the ChDIW technique were evaluated to address a major challenge in our technological world: electromagnetic interference (EMI) shielding. The ever‐increasing rate of emerging advanced electronics has led to the massive radiation of electromagnetic waves which not only is detrimental to human health but also negatively impacts sensitive electronics due the electromagnetic interference occurrence.^[^ ^26^, ^27^ ^]^ Electromagnetic (EM) waves interact with a shielding material through three different possibilities: reflection, absorption, and multiple reflections. The initial interaction is reflection that occurs due to the impedance mismatch between the conductive components within the shield and the free space.^[^ ^28^, ^29^, ^30^, ^31^ ^]^ However, because the reflection produces a secondary source of EM waves, it is not a favorable mechanism. The key target of an EMI shielding material should be absorption by which the EM waves are dissipated in the form of heat upon their interaction with the shielding material.

Multiple internal reflections or internal scattering is another shielding mechanism that can occur owing to the morphological and materials' design of some EMI shields. Numerous internal interfaces between materials with impedance mismatch or high porosity within structures such as cryogels majorly contribute to the internal scattering. In this regard, pores and/or the numerous interfaces within an engineered structure provide multiple back‐and‐forth reflections as the wave experiences mismatched impedances at each interface or pore wall. This can significantly prolong the propagation pathway of the EM waves prior to transmission, enhancing the likelihood of wave absorption.^[^ ^26^, ^28^, ^32^, ^33^, ^34^, ^35^, ^36^ ^]^ Considering this phenomenon, the chaotically printed cryogel in this work could be a promising candidate for boosting the internal scattering mechanism for the EMI shielding, leading to a high absorption. This beneficial property stemmed from the unique internal architecture of filaments produced by integrating conductive and magnetic materials through the chaotic advection. Aside from the favorable large interface area, incorporating both magnetic and electrically conductive features into one EMI shielding structure could record tremendously high absorption of EM waves.

**Figure 6:**
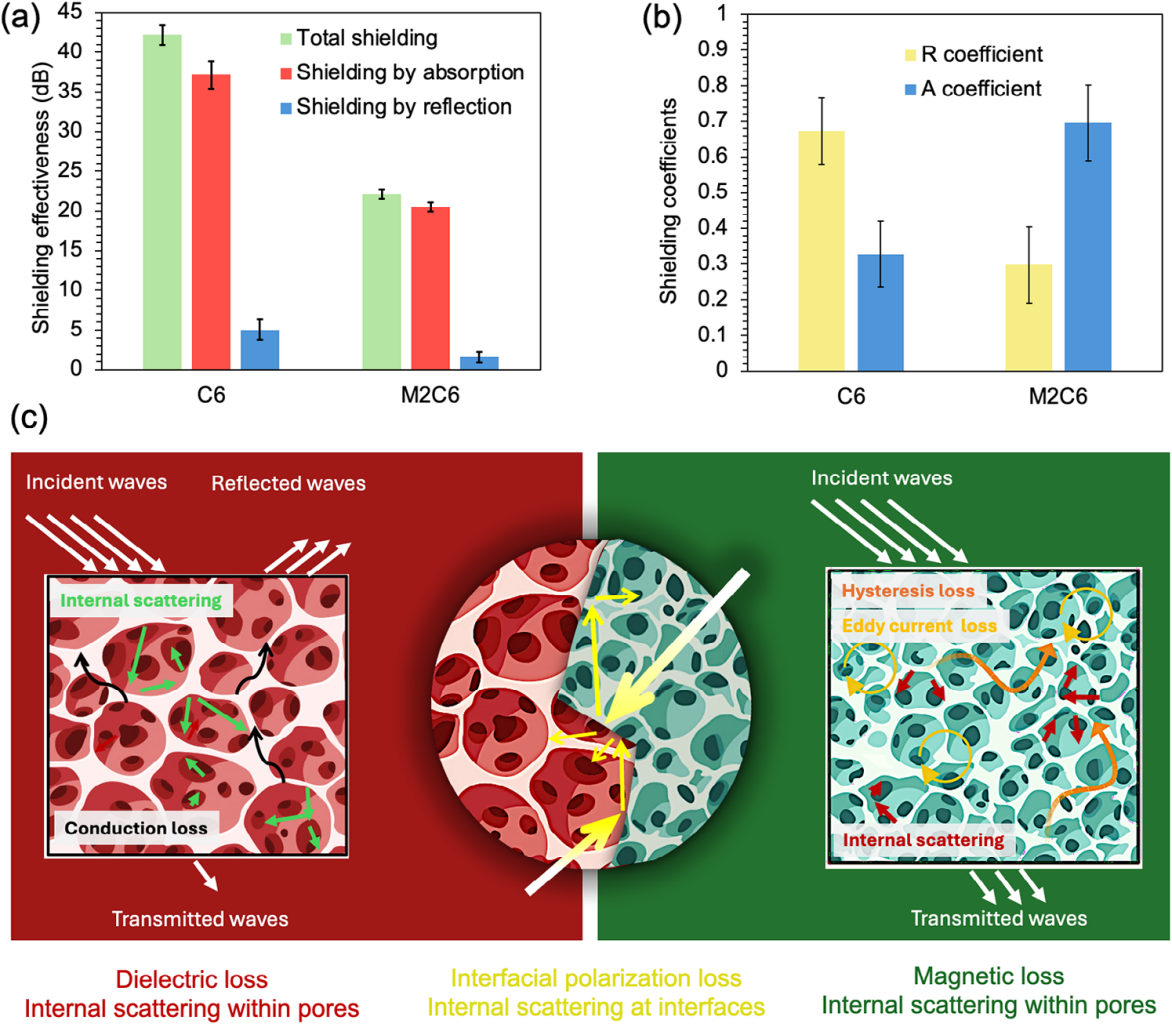
a,b) EMI shielding performance of the chaotically printed magnetic/conductive cryogel (M2C6) using the ChDIW approach compared to the purely conductive printed cryogel (C6) using the DIW technique: a) EMI shielding effectiveness (average SE_T_, SE_A_, and SE_R_ over the X‐band frequency range) and b) absorption and reflection coefficients of the pure conductive printed and the hybrid chaotically printed cryogels range (sample thickness = 5 mm). Error bars represent mean ± SD (*n* = 200). c) Mechanisms involved in the EMI shielding performance within the chaotically printed cryogel. Created in BioRender. Samsami, S. (2025) BioRender.com/y71c285.

To examine the capability of the chaotically printed cryogel to achieve absorption‐dominant behavior, the EMI shielding characteristics of this cryogel were evaluated. The respective formulations and parameters are discussed in the Supporting Information and Experimental section. First, a 3D‐printed cryogel composed of pure C6 ink was tested as the control specimen to have an accurate estimation of the effect of introducing our unique internal microstructure to the chaotically printed cryogel. The C6 cryogel demonstrated a very high total shielding effectiveness of 42.3 dB over the X‐band frequency range, as represented in **Figure** **6**a. Nonetheless, it is pivotal to highlight that the high conductivity of this structure led to a high reflection coefficient (R) of 63% and a corresponding absorption coefficient (A) of 37% (see Figure 6b). As discussed previously, this high reflectance causes secondary pollution, an inarguable challenge that our efforts were directed toward resolving by introducing a novel multilayered cryogel. The bulk cryogel created from chaotically extruded filaments, exhibited exceptional enhancement in the absorption coefficient, as represented in Figure 6b. This unique cryogel showed an outstanding absorption coefficient of 71% while exhibiting a satisfactory total shielding effectiveness of 22.1 dB. This means that the multilayered structure shielded 99.4% of the incident EM waves, 71% of which were absorbed. This exceptional behavior could be attributed to the several back‐and‐forth reflections within the multilayered shielding structure, arising from the vast area of interfaces between the alternating magnetic/conductive layers inside the cryogel. Besides, by endowing the shielding material with magnetic properties, the final cryogel was capable of efficiently absorbing the EM wave since the magnetic component (M2 ink) contributed to magnetic loss, while the conductive component (C6 ink) contributed to dielectric loss (see Figure 6c). The very low electrical conductivity of porous magnetic layers generated an adequate impedance match with the free space, allowing the EM waves to penetrate the cryogel bulk.^[^ ^37^, ^38^ ^]^ Consequently, the penetrated EM waves were dissipated and absorbed through the internal scattering due to the match/mismatch characteristics of alternating magnetic/conductive layers. This unique absorption‐dominant mechanism not only shields human and sensitive electronics from EM waves but also lessens the detrimental secondary EMI pollution.

To emphasize the supremacy of the structural design of the chaotically printed EMI shield, the analogous structures reported in the literature were investigated. To date, there are only a few studies on employing multi‐material designs for 3D‐printed EMI shields by DIW. For instance, a dual‐needle 3D printing was operated to print a magnetic ink with one printhead and a conductive ink with another printhead to produce a directional EMI shielding structure. The reflectance coefficient of this structure was found to be 0.5.^[^ ^39^ ^]^ Similarly, a high absorption coefficient of 0.73 was achieved via DIW of three conductive inks with different conductivities by three printheads, providing a gradient impedance mismatch within the structure.^[^ ^3^ ^]^ Nonetheless, using multiple printheads may not be as energy‐, time‐, and cost‐efficient as applying only one printhead like the strategy adopted in the present study. Furthermore, the creation of interfaces, especially with large surface areas, between different materials with varying porosities inside each filament was the remarkable achievement of this study that has not been reported so far.

## 3 Conclusion

A hierarchical design, from the nano‐ to the macroscale, was proposed to fabricate unique multifunctional cryogels. In a nutshell, one‐step ChDIW process was introduced to develop hybrid 3D multilayered soft materials and cryogels with a unique micro‐ordered internal architecture in a straightforward manner. The micro‐design of the layers was controlled by the chaotic mixing, considering the chemical compatibility and laminar flow of both inks, as well as the number of KSM elements. On the other hand, the macro‐design was dictated by the DIW process by adjusting the close yield stress and viscoelastic properties of the inks. Then, the ChDIW‐printed structures were freeze‐dried to achieve multilayered cryogels. These unique cryogels possessed a large interface area between the alternating layers inside each filament while each material at each side of the interfaces held distinctive properties, e.g., conductive/magnetic, and porosity. Compared to the conventional 3D‐printed multi‐materials, these cryogels offer superior versatility, where the materials’ characteristics and the internal micro‐pattern can be adjusted independently. The potential of this construct was applied to address an important challenge in the EMI shielding field: absorption‐dominancy. Benefiting from both magnetic and dielectric losses, as well as the multiple internal reflections arising from the large inner interfaces, the multilayered EMI shield showed an excellent absorption‐dominant mechanism with a high absorption coefficient of 0.71 and appreciable total shielding effectiveness (22.1 dB). This finding illustrated just one of the many potential applications of this cryogel. The incredible flexibility in controlling the nano‐ to the macroscale designs opens up exciting possibilities for diverse applications where interfaces and multifunctionality are of great importance, such as oil‐water separation, energy storage, carbon capture, wearable electronics, and photocatalysis.

## 4 Experimental Section

### Chemicals and Materials

Multi‐walled carbon nanotube (MWCNT, NC7000, purity >90%) with a dimension of 9.5 nm average diameter and 1.5 µm average length was purchased from Nanocyl to prepare the conductive gels. For the magnetic gels, iron (II, III) oxide (Fe_3_O_4_, purity 97%) with 50–100 nm average particle size was acquired from Sigma‐Aldrich. Cellulose nanocrystal (CNC) by CelluForce with average diameters of 2–5 nm and lengths of ≈100 nm was also used for cellulose‐based conductive and magnetic gel preparation.

### Preparation and Characterizations of Gels—Conductive Gel Preparation

Three different aqueous suspensions of CNC/MWCNT with the same mass ratio of 1:1 but different total solid contents (4, 6, and 8 wt%) were prepared as follows. First, MWCNT powder with 4, 6, and 8 wt% concentrations was added to deionized (DI) water and vortexed. Then, water suspensions of CNC with 4, 6, and 8 wt% concentrations were prepared separately. Afterward, 20 mL of the stable and well‐dispersed CNC suspensions were added to 20 mL of the corresponding MWCNT aqueous suspensions and then probe sonicated by Qsonica Misonix XL‐ 2000 series sonicator. All suspensions were sonicated for 20 min with 5 min time intervals. C_4_ gel denotes 4 wt% of CNC and 4 wt% of MWCNT; likewise, C_6_ and C_8_ gels were obtained (see Table S1, Supporting Information). In the end, all the gels were processed with a homogenizer (Fisher Scientific 850 homogenizer) at 7000–8000 rpm for 5 min to ensure homogeneous dispersion of the components. It should be noted that the reason behind keeping the mass ratio at 1:1 for all suspensions was that, based on the existing studies in the literature,^[^ ^40^ ^]^ the equal mass content of CNC and MWCNT is the most stable and well‐dispersed colloid in water.

### Preparation and Characterizations of Gels—Magnetic Gel Preparation

Three different aqueous suspensions of CNC/Fe_3_O_4_ with different mass ratios of 1:0.25, 1:0.5, and 1:0.75 (CNC to Fe_3_O_4_) were prepared in the following manner. First, water suspension of CNC with 30 wt% concentration was prepared. Then, Fe_3_O_4_ powders with 22.5, 15, and 7.5 wt% concentrations were added to DI water separately and vortexed. Subsequently, 20 mL of the stable and well‐dispersed CNC suspension was added to 20 mL of each aqueous suspension of Fe_3_O_4_ separately followed by vortexing. Later, all the gels were homogenized with a homogenizer at 7000–8000 rpm for 3 min to ensure homogeneous dispersion of the magnetic nanoparticles. The gel with a mass ratio of 1:0.25 (CNC to Fe_3_O_4_) was labeled as M1. As described in Table S1 (Supporting Information), M2, and M3 gels denote CNC to Fe_3_O_4_ mass ratios of 1:0.5 and 1:0.75, respectively.

### Preparation and Characterizations of Gels—Rheological Properties

The rheological parameters of the gels are fundamentally related to the processes of 3D printing and must be measured properly to determine the optimal printing conditions. The rheological parameters were conducted by a rheometer (Discovery HR20, TA Instruments) equipped with 20 mm parallel plate geometries and a gap size of 1 mm. To investigate the flow behavior of gels, a steady‐state shear test in which a shear rate ranged from 0.1 to 500 s^−1^ was exploited. Afterward, strain sweep and frequency sweep tests were performed to determine the viscoelastic properties of both magnetic and conductive gels. The linear viscoelastic region was ascertained based on the strain amplitude sweep tests for all gels. To carry this out, strain amplitudes ranged from 0.1 to 1000% at a constant angular frequency of 1 rad s^−1^. Subsequently, the frequency sweep test was accomplished in the linear viscoelastic region with a frequency range of 0.1 to 100 rad s^−1^ and a constant strain amplitude of 1%. All tests were conducted at room temperature. Subsequently, a rheometer (Kinexus, Netzsch) equipped with 20 mm parallel plate geometries and a gap size of 1 mm was supplied to carry out the creep tests for accurately measuring the yield stress of the optimal gels.

### Preparation and Characterizations of Gels—Electrical Conductivity and Magnetic Properties Measurements

The Ossila four‐point probe was used to determine the conductivity of the optimal conductive ink. A vacuum filtration setup including a Buchner funnel with a filter paper and a filter flask connected to a vacuum pump was utilized to obtain a thin film of the material with 18 mm diameter and microscale thickness (see Figure 4a_2_). The measurements were conducted at four different points on the sample, and the average value was reported.

The magnetic properties of the optimal magnetic ink and pure Fe_3_O_4_ nanoparticles were measured by a Vibrating Sample Magnetometer (8600 Series VSM, Lake Shore Cryotronics) with EM4‐CSB magnet, LS643 power supply, and 86‐LC coil set. The coercivity and saturation magnetization were acquired from the hysteresis loops. The dried magnetic sample was ground by a mortar to prepare the powder for the analysis.

### 3D Printing of Inks

A 3D bioprinter (Bio X, Cellink) equipped with 10 mL syringes was used to deposit the CNC‐based inks. The flow of ink could be adjusted by setting suitable pressure and printer speed based on the different ink formulations and their rheological properties. Finally, conductive samples were frozen by being placed in liquid Nitrogen for about 2 min. And, the magnetic 3D printed samples were kept in a freezer for 24 h. Thereafter, these frozen samples were lyophilized at 0.005 mbar using a freeze dryer (LabConco, 2.5 liters) for 24 h to obtain the corresponding cryogels.

### ChDIW Technology

The chaotic system was composed of a syringe pump for delivering inks into the Kenics static mixing (KSM) printhead with a nozzle tip to co‐extrude the inks and then shape them with the aid of a 3D printer. The two conductive and magnetic inks were dispensed through silicone tubing into the 2KSM printhead at a constant rate (0.5 mL min^−1^) controlled by a syringe pump (pump 33 DDS, dual drive system). A 13G nozzle tip was used to deposit the co‐extruding inks on a glass slide with the aid of a 3D bioprinter described before (printing speed = 6 mm s^−1^). Finally, the chaotically 3D‐printed sample was frozen by being placed in liquid Nitrogen for about 2 min followed by lyophilization for 24 h to obtain the resultant cryogel.

### ChDIW Technology—Fluorescence Microscopy

To evaluate the creation of interfaces within the internal structure of the filaments, fluorescence microscopy analysis was applied. First, the C6 conductive and M2 magnetic inks were dyed by adding commercial green (Fluor Green 5404) and red (Fluor Hot Pink 5407) fluorescent dyes (Createx Colors, East Granby, CT, USA), respectively. After performing the chaotic DIW, the final printed structure was frozen by being placed in liquid Nitrogen for about 1 min and the cross‐sectional cuts were made instantly on it. Nikon Eclipse Ts2R microscope equipped with a 4x objective lens was utilized for imaging from the filaments.

### ChDIW Technology—Morphology

The microstructure and morphology of 3D‐printed structures play essential roles in advanced applications. Here, scanning electron microscopy (SEM, Axia ChemiSEM, Thermo Scientific) was applied to investigate the conductive cryogel's morphology. The magnetic and chaotically printed cryogels were first coated by a thin layer of gold using a sputter coater machine (Polaron Instruments Inc.) to reduce charge interruptions. Next, a Tescan Vega3 SEM was utilized to take images from these specimens. In addition, energy dispersive spectroscopy (EDS) was performed on the chaotic 3D‐printed cryogel to study the formed interfaces between layers within each filament. To carry this out, the Tescan Vega3 SEM equipped with an EDS detector (PentaFET Precision, Oxford Instruments) was used.

### ChDIW Technology—EMI Shielding Performance Measurements

To evaluate the EMI shielding properties within the X‐band frequency range of 8.2‐12.4 GHz, a Keysight vector network analyzer (VNA) equipped with a rectangular waveguide was employed. The cryogel samples with 5 mm thickness were placed between the waveguide adaptors that were connected to the VNA by microwave cables. The shielding parameters were calculated using S‐parameters. While S_21_ and S_12_ indicate the transmission of incident voltage magnitude at ports 1 and 2, S_11_ and S_22_ represent the reflection of incident voltage magnitude at the ports. Total shielding effectiveness (SE_T_), reflection loss (SE_R_), and absorption loss (SE_A_) values of samples were determined using the following equations:^[^ ^26^, ^41^, ^42^ ^]^

(1) $$SE_{R}=10\;\log\;\frac{1}{1-R}=10\;\log\;\frac{1}{1-|S_{11}{|}^{2}}$$

(2) $$SE_{A}=10\;\log\;\frac{1-R}{T}=10\;\log\;\frac{1-|S_{11}{|}^{2}}{|S_{21}{|}^{2}}$$

(3) $$SE_{T}=SE_{R}+SE_{A}$$

(4) A+R+T=1

where *A*, *R*, and *T* are absorption, reflectance, and transmittance coefficients, respectively.

More information regarding the formulas used for calculating the EMI shielding parameters is discussed in the Supporting Information file.

## Conflict of Interest

The authors declare no conflict of interest.

## Author Contributions

S.S., M.K., and K.C.T. conceived the project. S.S. conducted the experimental work and wrote the manuscript. Z.M.K. assisted with magnetic ink synthesis and characterizations. J.F.Y.‐dL. and D.A.Q.M. assisted with the design and fabrication of chaotic nozzles. G.T.‐dS. and M.M.A. oversaw the project in terms of chaotic printing. K.C.T. and M.K. supervised the overall project. All authors discussed the results and made contributions to the manuscript.

## Supporting information



Supporting Information

Supplemental Video 1

Supplemental Video 2

Supplemental Video 3

## Data Availability

The data that support the findings of this study are available from the corresponding author upon reasonable request.
