# Supplementary material for: Chaotic Direct Ink Writing (ChDIW) of Hybrid Hydrogels: Implication for Fabrication of Micro‐ordered Multifunctional Cryogels
Source: Small Methods. 2025 Mar 13;9(8):2500349. doi: 10.1002/smtd.202500349 (PMC12391654; doi:10.1002/smtd.202500349)
Supplement: Supplementary file 1 — Supporting Information [file SMTD-9-2500349-s001.docx]

**Chaotic Direct Ink Writing (DIW) of Hybrid Hydrogels: Implication for Fabrication of Micro-ordered Multi-functional Cryogels**

Shakiba Samsami ^a^, Zahra Monsef Khoshhesab ^a^, Juan Felipe Yee-de León ^b^, Diego Alonso Quevedo Moreno ^c^, Mario Moisés Alvarez ^b^, Grissel Trujillo-de Santiago ^b^, Kam C Tam *^a^, Milad Kamkar *^a^

*^a^ Department of Chemical Engineering, University of Waterloo, Waterloo, Ontario, N2L 3G1, Canada*

*^b^ Departamento de Ingeniería Mecatrónica, Tecnologico de Monterrey, Monterrey, NL, 64849, Mexico*

*^c^ Department of Mechanical Engineering, Massachusetts Institute of Technology, Cambridge, MA 02139, USA*

Table of Contents

[Preliminary Assessments 3](#_Toc188355658)

[Conductive and Magnetic Gels Preparation 4](#_Toc188355659)

[Conductive Hydrogels Optimization 5](#_Toc188355660)

[Magnetic Hydrogels Optimization 7](#_Toc188355661)

[Conductive and Magnetic Gels Printability 9](#_Toc188355662)

[Dispersity of Fe_3_O_4_ Nanoparticles within the Optimized M2 Hydrogel 10](#_Toc188355663)

[Multi-functional Structure by the ChDIW Technique 11](#_Toc188355664)

[EMI Shielding Effectiveness 14](#_Toc188355665)

[References 16](#_Toc188355666)

## **Preliminary Assessments**

To preliminary examine the ability of paste-like gels to create multilayered filaments, 15 wt% CNC hydrogels were applied to KSM printheads. To ensure the laminar flow of the CNC hydrogels through the KSM printheads for a successful micro-scale design, their Reynolds numbers were calculated. First, the shear rate experienced by inks during the ChDIW process was calculated via Eq. 1 [1]:

$\text{γ˙}$= $\frac{\text{4Q}}{\text{π}\text{R}^{\text{3}}}$ Eq. 1

$\text{γ˙}_{\text{without needle}}$= $\frac{\text{4}\text{ }\text{×}\text{ }\text{(1.5 mL/min)}}{\text{π}{\text{ }\text{×}\text{ }\text{(}\text{1 m}\text{m)}}^{\text{3}}}$ = $\frac{\text{4}\text{ }\text{×}\text{ }\text{1.5 }\text{cm}^{\text{3}}\text{/min}}{\text{π}{\text{ }\text{×}\text{ }\text{ }\text{0.001}\text{cm}}^{\text{3}}}$ = 1909.85 1/min = 31.8 1/s

$\text{γ˙}_{\text{14G}}$ = $\frac{\text{4}\text{ }\text{×}\text{ }\text{(1.5 mL/min)}}{\text{π}{\text{ }\text{×}\text{ }\text{(0}\text{.}\text{75 mm)}}^{\text{3}}}$ = $\frac{\text{4}\text{ }\text{×}\text{ }\text{1.5 }\text{cm}^{\text{3}}\text{/min}}{\text{π}{\text{ }\text{×}\text{ }\text{0.0004}\text{2}\text{ cm}}^{\text{3}}}$ = 4547.28 1/min = 75.78 1/s

$\text{γ˙}_{\text{15G}}$ = $\frac{\text{4}\text{ }\text{×}\text{ }\text{(1.5 mL/min)}}{\text{π}{\text{ }\text{×}\text{ }\text{(0.715 mm)}}^{\text{3}}}$ = $\frac{\text{4}\text{ }\text{×}\text{ }\text{1.5 }\text{cm}^{\text{3}}\text{/min}}{\text{π}{\text{ }\text{×}\text{ }\text{0.00036 cm}}^{\text{3}}}$ = 5305.16 1/min = 88.42 1/s

where Q and R represent the flow rate of the flowing inks and the radius of the needle connected to the KSM printhead, respectively. For the first test (without any needle), the radius was considered equal to the outlet radius of the KSM printhead.

Next, the corresponding viscosities of CNC inks at these particular shear rates were obtained from their flow curves depicted in **Figure S1a**: $\eta_{\text{without needle}}$ ≃ 7.21 Pa.s, $\eta_{\text{14G}}$ ≃ 3.75 Pa.s, and $\eta_{\text{15G}}$ ≃ 3.5 Pa.s. As expected, needles with lower diameters applied higher shear rates on the inks, leading to decreased viscosity of the CNC hydrogel due to its shear-thinning behavior.

Subsequently, the Reynolds number of CNC hydrogels was calculated by Eq. 2. The density ($\text{ρ}$) of the prepared inks was obtained experimentally. It is notable that the generalized Reynolds number for a shear-thinning fluid can be calculated accurately by models such as the Ostwald model [2]. However, for simplification, we used Eq. 2 here to just assess the laminarity of our gels under the printing conditions in the syringe.

Re = $\frac{\text{ρvD}}{\eta}$ = $\frac{\text{ρ}\text{ }\text{×}\text{ }\text{(}\frac{\text{Q}}{\text{A}}\text{)}\text{ }\text{×}\text{ }\text{2R}}{\eta}$ = $\frac{\text{ρ}\text{ }\text{×}\text{ }\text{(}\frac{\text{Q}}{R\text{π}}\text{)}\text{ }\text{×}\text{ }\text{2}}{\eta}$ = $\frac{\text{2Qρ}}{\text{η}\text{Rπ}}$ Eq. 2

$\text{Re}_{\text{without needle}}$ = $\frac{\text{2 × (}\frac{\text{1.5}}{\text{60}}\text{ }\text{cm}^{\text{3}}\text{/s) × (1.19 g/}\text{cm}^{\text{3}}\text{) }}{\text{(7.21 g/mm.s) }\text{× (1 mm) × π}}$ ≃ 0.0026

$\text{Re}_{\text{14G}}$ = $\frac{\text{2 × (}\frac{\text{1.5}}{\text{60}}\text{ }\text{cm}^{\text{3}}\text{/s) × (1.19 g/}\text{cm}^{\text{3}}\text{) }}{\text{(3.75 g/mm.s) }\text{× (0.75 mm) × π}}$ ≃ 0.0067

$\text{Re}_{\text{15G}}$ = $\frac{\text{2 × (}\frac{\text{1.5}}{\text{60}}\text{ }\text{cm}^{\text{3}}\text{/s) × (1.19 g/}\text{cm}^{\text{3}}\text{) }}{\text{(3.5 g/mm.s) }\text{× (0.715 mm) × π}}$ ≃ 0.0076

As determined, the CNC 15 wt% inks owned very low Reynolds numbers, confirming their laminar flow. Thus, these inks could flow side by side through the KSM printheads to generate well-ordered microlayers.


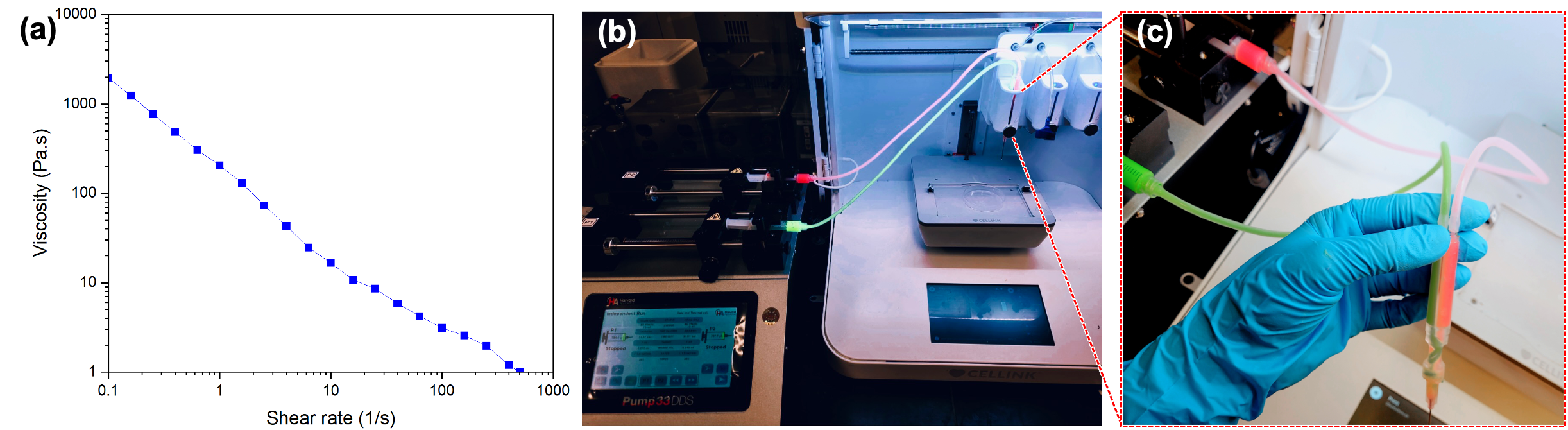


**Figure S1.** (a) Flow curve of CNC 15 wt% hydrogel. (b) The ChDIW setup consisting of a 3D printer and (c) a syringe pump for delivering hydrogel inks into the 2KSM printhead.

## **Conductive and Magnetic Gels Preparation**

Three magnetic and three conductive CNC-based hydrogels were prepared systematically as described in the material and methods section. For the conductive inks, the mass ratio of CNC to MWCNT was constant for all gels, and only the total solid content differed. For magnetic gels, different mass ratios of Fe_3_O_4_ to CNC were considered. The detail of each gel’s formulation is given in **Table S1**.

**Table S1.** Summary of material concentrations utilized in the preparation of the conductive and magnetic suspensions. The optimal formulations are highlighted in yellow color.

| Sample ID | CNC content (wt%) | MWCNT content (wt%) | Fe_3_O_4_ content (wt%) | Note |
| --- | --- | --- | --- | --- |
| C4 | 4 | 4 | 0 | Insufficient layer support |
| C6 | 6 | 6 | 0 | Excellent printability |
| C8 | 8 | 8 | 0 | Needle clogging |
| M1 | 15 | 0 | 3.75 | Printable |
| M2 | 15 | 0 | 7.5 | Excellent printability |
| M3 | 15 | 0 | 11.25 | Needle clogging |

## **Conductive Hydrogels Optimization**

*Chemistry*

CNCs are generally dispersed well in water due to the hydrogen bonds formation between their abundant hydrophilic hydroxyl groups and water molecules. Quite the reverse, the (2 0 0) plane of cellulose is highly hydrophobic since it displays mostly C-H bonds [3, 4]. This amphiphilic characteristic of CNCs caused good dispersion of MWCNTs within a uniform CNC/MWCNT aqueous suspension. Contrary to the non-homogenous C8 suspension, the MWCNTs were finely dispersed in the C4 and C6 suspensions without any visible agglomeration due to the assistance of CNCs by adjusting the interfacial interactions. Before delving into these interactions, as an essential prerequisite for guaranteeing interactions between CNCs and MWCNTs, ultrasonication disrupted the aggregations between MWCNTs caused by π–π stacking via the sp^2^ carbon lattice. As illustrated in **Figures 1b** and **d**, with the aid of ultrasonication, CNCs were then driven toward the carbon nanotubes and localized on their surface, inducing the hydrophobic effect between MWCNTs and CNCs [5-8]. Particularly, in our case, CNCs had excellent colloidal stability in water because of their negatively charged sulfate half-ester groups.

*Rheological Behavior*

Essentially, the flow behavior and viscoelastic properties of gels are the main characteristics of any extrusion-based DIW process. Considering the flow behavior, the gels must show shear-thinning behavior to be extruded through a narrow nozzle under shear stress. **Figure S2a** displays that all the prepared conductive suspensions showed shear-thinning behavior. The C4 suspension displayed a so-called three-region shear-thinning behavior including the shear-thinning behavior at low and high shear rates, and Newtonian behavior at intermediate shear rates. This can be attributed to the fact that this suspension was in the liquid crystal regime, and the disassociation of liquid crystal clusters into individual crystals occurs at intermediate shear rates followed by their subsequent alignment along the shear direction [9-11]. The C6 suspension exhibited only a single shear-thinning region since it was in the gel regime. This was while the C8 suspension did not show a typical shear-thinning flow behavior because of its non-homogeneity structure. When shear stress was applied, some areas deformed easily while others resisted flow, creating a complex flow behavior. In addition, the applied shear stress began to unravel the high content of entangled MWCNT in this suspension. Once enough shear stress was applied, this structure collapsed through a steep drop in viscosity as no reliable interactions between CNCs and MWCNTs had been formed before.

What matters after the deposition of the materials is that the deformation should be minimized to ensure shape fidelity. To investigate this aspect, the viscoelastic properties of suspensions were studied. **Figure S2b** depicts the loss (G′′) and storage (G′) moduli versus strain amplitude to distinguish the linear and nonlinear viscoelastic regions of the suspensions. Based on the results, the strain amplitude of 1% was designated as a safe value for all samples to do the frequency sweep test within the linear viscoelastic region. Consequently, the findings for the frequency sweep test represented in **Figure S2c** confirmed that the storage modulus was noticeably higher than the loss modulus in the entire frequency range for all conductive suspensions. Besides, both dynamic moduli showed a frequency-independent trend, showing the gel-like behavior for all suspensions.

## **Magnetic Hydrogels Optimization**

*Chemistry*

The interaction between CNCs and Fe_3_O_4_ nanoparticles within the magnetic gel predominantly occurred between the hydroxyl groups on CNCs and the magnetic nanoparticles by forming coordinate bonds. Additionally, the –OH groups on CNCs could form hydrogen bonds with oxygen atoms on the Fe_3_O_4_ surface, enhancing the immobilization of magnetic nanoparticles in the cellulose matrix [12]. **Figures 1b** and **d** schematically describe what possibly took place in the magnetic suspensions in terms of how the components interacted.

*Rheology and Printability*

Rheology tests were conducted to quantify how the gels would flow and deform under different conditions while 3D printing. **Figure S2d** represents that all prepared magnetic suspensions featured shear-thinning behavior, ensuring a smooth flow through the printing needle. It is worth pointing out that all magnetic suspensions containing 15 wt% CNC (much higher CNC content compared to the conductive inks) showed a single shear-thinning flow behavior since they were absolutely in the gel regime. Regarding the viscoelastic properties, consistent with the strain sweep test results indicated in **Figure S2e**, the strain amplitude of 1% was selected for performing the frequency sweep test on all samples. Accordingly, the dynamic moduli of all magnetic suspensions possessed gel-like behavior in the entire frequency window as depicted in **Figure S2f**. It is worth revealing that all magnetic suspensions displayed a weak strain overshoot in the strain sweep tests due to the presence of Fe_3_O_4_ nanoparticles as crosslinkers between CNCs, forming weak microstructures that resist flow alignment [13, 14]. This phenomenon also contributed to a steep slope in the dynamic modulus of gels over the non-linear region because they became stiffer. Furthermore, it is worthy of note that the magnetic gels showed a shallower slope in dynamic modulus over the linear region compared to the conductive gels. This could be attributed to the flexibility of MWCNTs by sliding past each other under increasing strain.

**
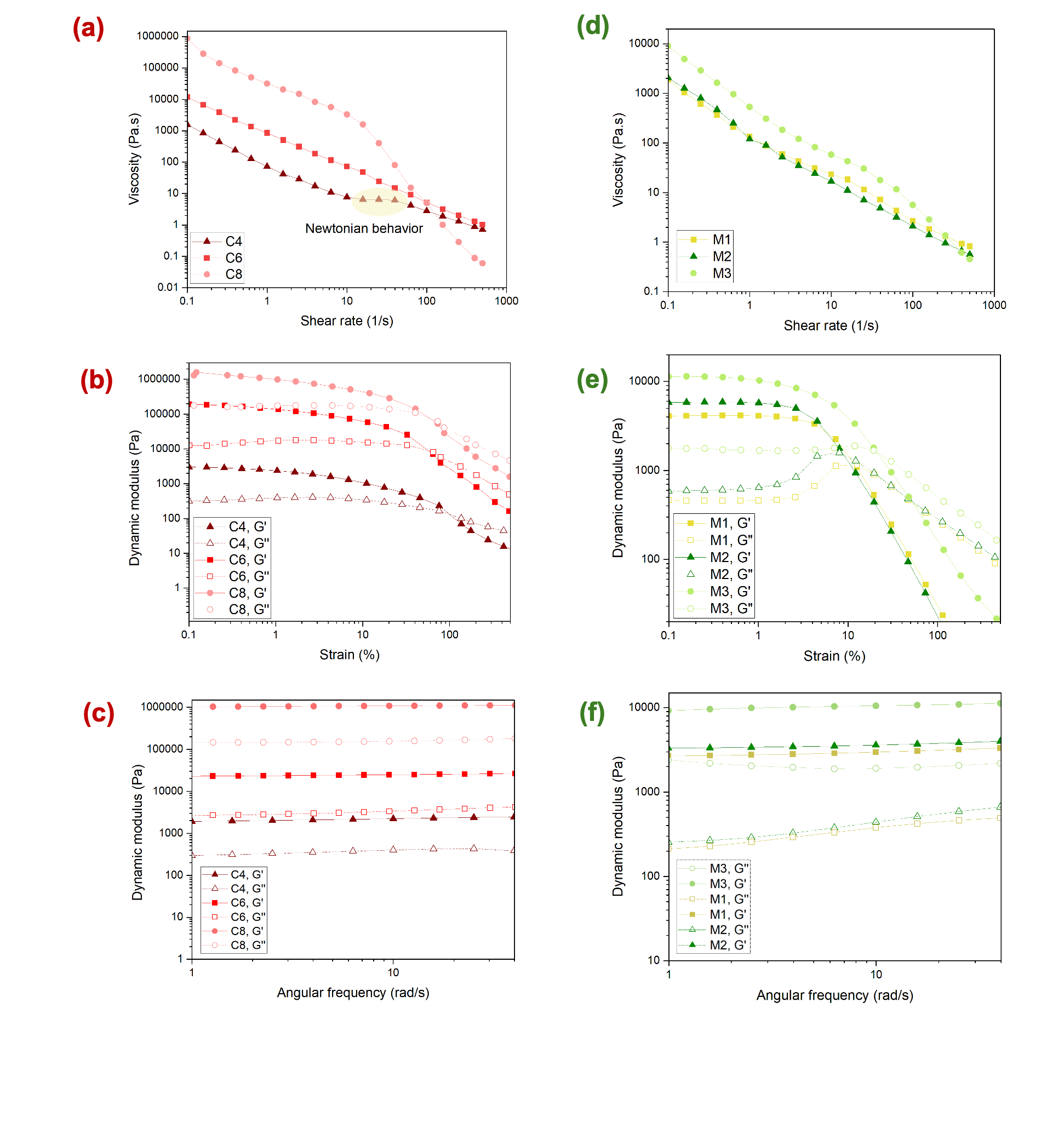
**

**Figure S2.** The rheological characterizations of the (a-c) conductive and (d-f) magnetic gels: viscosity of the prepared gels versus shear rate (a and d), storage and loss modulus of the gels versus strain amplitude (b and e), and angular frequency (c and f).

## **Conductive and Magnetic Gels Printability**

**Figure S3** shows the printed structures by all the prepared conductive and magnetic gels. Considering the conductive gels, the C4 and C8 hydrogels were not printable because of insufficient support layer and nozzle clogging due to non-homogeneity, respectively. The C6 sample showed excellent printability, so it was selected as the optimal conductive ink in this work. Considering the printability of magnetic gels, **Figure S3** demonstrates that the M1 and M2 gels were printable while the M3 gel caused needle clogging due to its high viscosity. Comparing 3D structures printed by the M1 and M2 gels, the M2 gel provided higher shape accuracy and printing quality. The green circles on the printed structure by M1 gel show its lower printing accuracy compared to the M2 gel.

**Figure S3.** Photographs of the 3D-printed structures by the conductive (DIW conditions: pressure = 50 kPa, speed = 12 mm/s, needle size = 18G) and magnetic (DIW conditions: pressure = 70 kPa, speed = 12 mm/s, needle size = 25G) gels.

## **Dispersity of Fe_3_O_4_ Nanoparticles within the Optimized M2 Hydrogel**

The microstructure of any 3D printed structure generally has a critical role in advanced applications. Plus, it could be beneficial here for probing how well the Fe_3_O_4_ nanoparticles were dispersed within the CNC hydrogel. With this perspective, **Figure S4** shows that Fe_3_O_4_ nanoparticles were well-dispersed all over the structure without any noticeable aggregation to hinder the interactions between the CNCs and Fe_3_O_4_ nanoparticles.


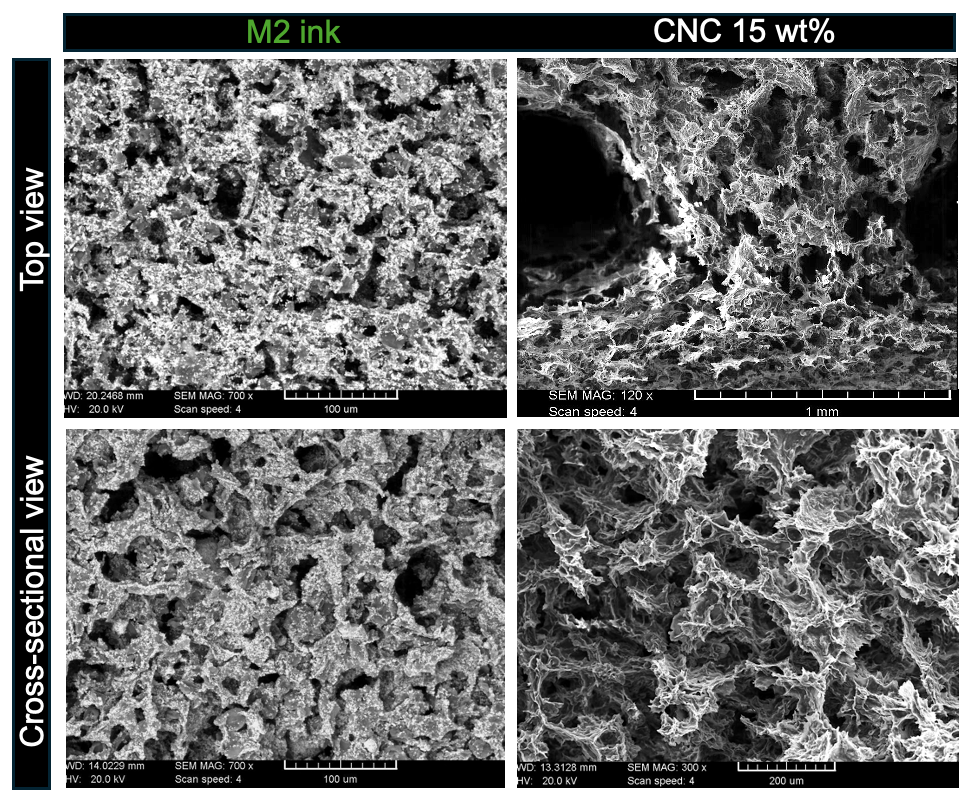


**Figure S4.** SEM images of the top and cross-sectional views of the M2 and pure CNC 15 wt% cryogels, showing the homogeneous dispersion of Fe_3_O_4_ nanoparticles within the M2 gel structure.

## **Multi-functional Structure by the ChDIW Technique**

**Figures S5a** and **b** show the setup used for conducting the hybrid ChDIW process composed of the 2KSM printhead fed up with the optimized conductive and magnetic inks via the syringe pump applied to a 3D printer. The chaotically printed sample shown in **Figure S5c** confirmed the high co-extrudability and co-printability of C6 and M2 optimized inks.


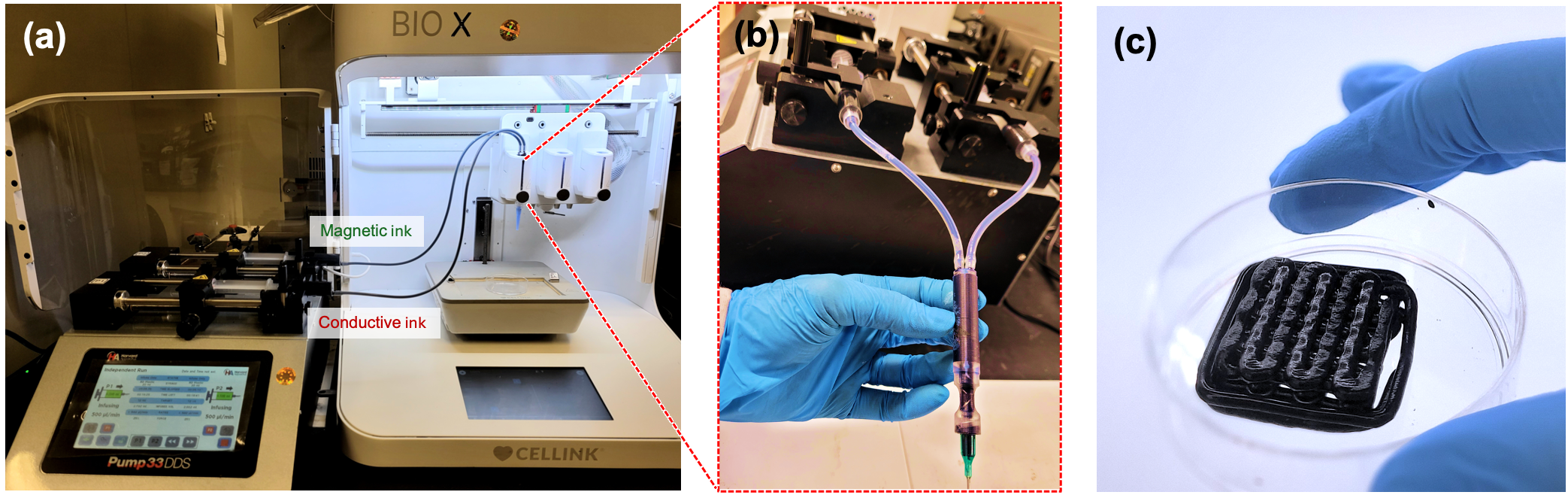


**Figure S5.** (a) The ChDIW setup consisting of a 3D printer and (d) a syringe pump for delivering M2 and C6 inks into the 2KSM printhead. (c) Chaotically printed sample containing M2 and C6 inks.

To ensure the laminar flow of the conductive and magnetic inks through the 2KSM printhead, their Reynolds numbers were calculated as described earlier. The shear rate experienced by both inks during the ChDIW process was calculated via Eq. 1:

𝛾˙ = $\frac{\text{4}\text{ }\text{×}\text{ }\text{(0.5 mL/min)}}{\text{π}{\text{ }\text{×}\text{ }\text{(1 mm)}}^{\text{3}}}$ = $\frac{\text{4}\text{ }\text{×}\text{ }\text{0.5 }\text{cm}^{\text{3}}\text{/min}}{\text{π}{\text{ }\text{×}\text{ }\text{0.001 cm}}^{\text{3}}}$ = 636.62 1/min = 10.61 1/s

Next, the corresponding viscosities of both inks at this specific shear rate were obtained from their flow curves presented in **Figure 4c_1_**: $\text{η}_{\text{M2}}$ = 16.8 Pa.s and $\text{η}_{\text{C6}}$= 73.52 Pa.s. Subsequently, the Reynolds numbers of both inks were calculated by Eq. 2. The density of the prepared inks was acquired experimentally.

$\text{Re}_{\text{M2}}$ = $\frac{\text{2 × (}\frac{\text{0.5}}{\text{60}}\text{ }\text{cm}^{\text{3}}\text{/s) × (1.22 g/}\text{cm}^{\text{3}}\text{) }}{\text{(16.8 g/mm.s) }\text{× (1 mm) × π}}$ ≃ 0.0004

$\text{Re}_{\text{C6}}$ = $\frac{\text{2 × (}\frac{\text{0.5}}{\text{60}}\text{ }\text{cm}^{\text{3}}\text{/s) × (1.14 g/}\text{cm}^{\text{3}}\text{) }}{\text{(73.52 g/mm.s) }\text{× (1 mm) × π}}$ ≃ 0.00008

As demonstrated, both optimized magnetic and conductive inks possessed very low Reynolds numbers, ensuring the laminar flow. Hence, it was concluded that these inks could flow side by side through the 2KSM printhead to generate well-ordered microlayers due to their guaranteed laminar flow.

After addressing the micro-design requirements, one important factor for assessing the macro-design was the ability of the hybrid material to not yield under the weight of the subsequent layers. In this regard, the maximum achievable height of the final 3D-printed multi-material without collapsing was calculated based on the material’s yield stress. One of the most precise techniques to determine the yield stress is conducting multiple creep tests that involve scanning the changes in the strain versus time under different applied stresses. **Figure S6** shows the test results of multiple creep tests utilized for finding the yield stress of the optimized C6 and M2 hydrogels. The yield stress of C6 and M2 hydrogels was found to be in the range of 150-200 and 250-300 Pa, respectively. Notably, the M2 gel exhibited an abrupt rise in the strain amplitude upon increasing the stress above the yield stress while the C6 gel showed a gradual increment in the strain amplitude. This behavior was in good agreement with the result of the strain sweep tests discussed before.


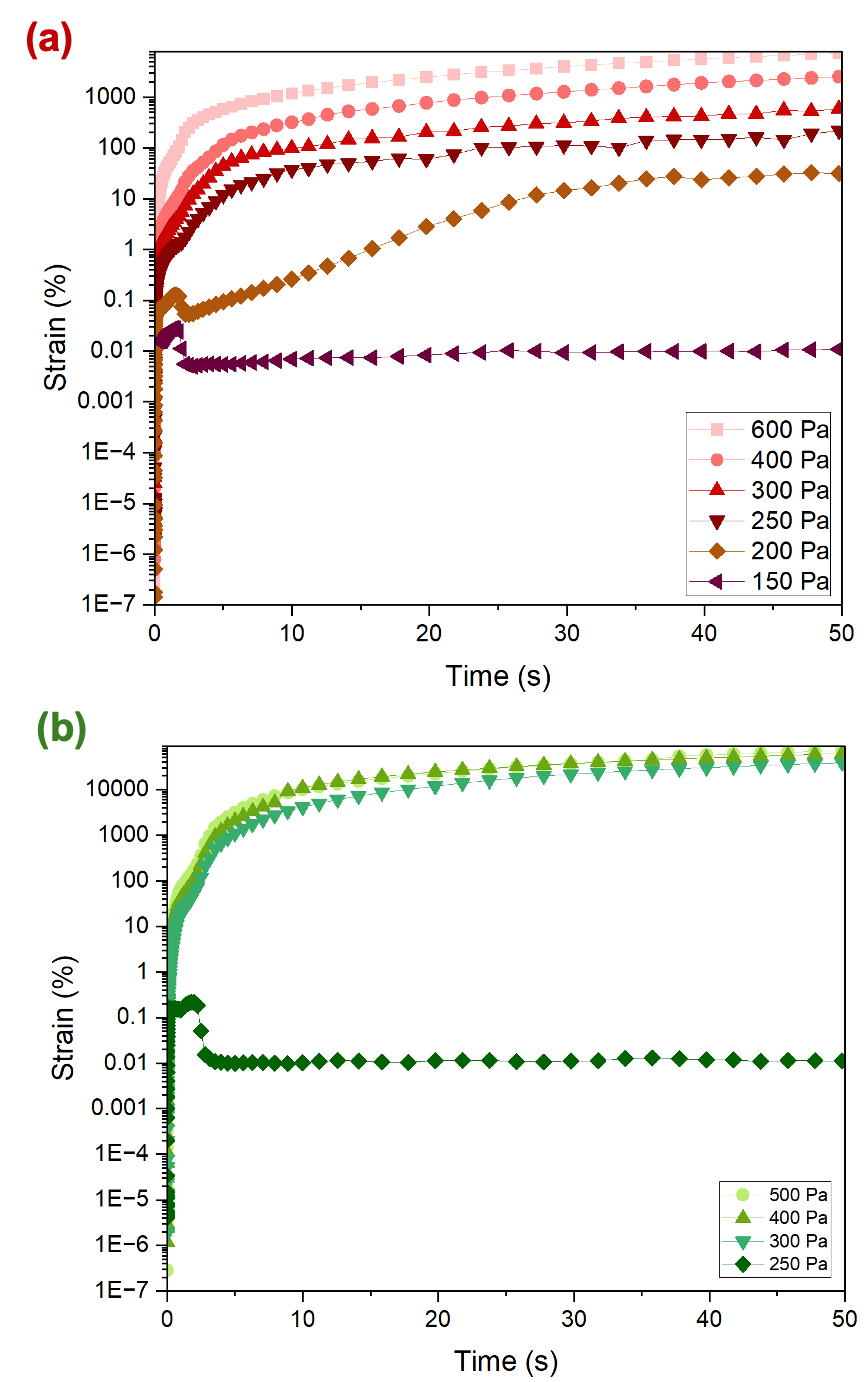


**Figure S6.** Determination of the yield stress of both optimized (a) conductive C6 and (b) magnetic M2 inks via multiple creep tests by applying different shear stresses.

The maximum achievable height (H) of the printed multi-material sample was calculated by Eq. 3 [15]. According to this equation, the lower yield stress ($\text{τ}_{\text{y}}$) of the C6 ink compared to the M2 ink was considered to ensure the accurate maximum endurable height by both inks. Also, the density of the multilayered filaments was estimated as the average densities of both inks since they contributed equally to the hybrid filament formation.

H = $\frac{\text{τ}_{\text{y}}}{\text{ρg}}$ Eq. 3

H = $\frac{\text{150}\text{ }\text{kg/}\text{m.}\text{s}^{\text{2}}}{\text{(1.18 }\text{×}\text{ }\text{10}^{\text{3}}\text{ k}\text{g/}\text{m}^{\text{3}}\text{) }\text{× (}\text{9.8}\text{1}\text{ m/}\text{s}^{\text{2}}\text{)}}$ = 0.0129 m ≃ 13 mm

Hence, it was revealed that the multilayered filaments could support approximately 13 mm of the subsequent layers. This finding firmly confirmed that the spreading of the filaments within our produced chaotically printed sample with 5mm height did not happen.

# **EMI Shielding Effectiveness**

In this study, a two-port Keysight vector network analyzer (VNA) was employed to characterize the shielding performance of the prepared cryogels. In this system, a single-frequency signal was sent toward the sample from port one (S_1_), and then the transmitted (S_21_) and reflected (S_11_) waves were recorded. The same process was conducted with the wave sent from port two. Then, this measurement was repeated over a range of frequencies (8-12 GHz). The S- parameter was used to measure the EMI shielding coefficients, including reflectance (R), and transmittance (T). The R and T coefficients which are the power of the reflected and transmitted waves, respectively, could be found from the following formulas [16, 17]:

$\text{R = }\frac{\text{P}_{\text{R}}}{\text{P}_{\text{I}}}\text{ = }\text{S}_{\text{11}}^{\text{2}}$ Eq. 4

$\text{T = }\frac{\text{P}_{\text{T}}}{\text{P}_{\text{I}}}\text{ = }\text{S}_{\text{21}}^{\text{2}}$ Eq. 5

The absorption coefficient (A), could also be calculated via the following equation [16, 17]:

A = 1 – R – T Eq. 6

The total shielding effectiveness (SE_T_) as a vital parameter for estimating the total shielding performance of an EMI shield was calculated via Eq. 7 [16, 18, 19]:

$\text{SE}_{\text{T}}\text{ = 10 log}\frac{\text{P}_{\text{I}}}{\text{P}_{\text{T}}}\text{ = 10 log }\frac{\text{1}}{\text{T}}\text{ = -10 log T }$= -10 log $\text{S}_{\text{21}}^{\text{2}}$ Eq. 7

Eqs. (8-10) have been developed to compute the contribution of absorption ($\text{SE}_{\text{A}}$), reflection ($\text{SE}_{\text{R}}$), and multiple-reflection ($\text{SE}_{\text{MR}}$) to the SE_T_ of a shielding material [16-20]. It is notable that $\text{SE}_{\text{MR}}$ could be neglected since the thickness of the specimen was greater than the skin depth of the wave. The skin depth is defined as the thickness beneath the shield's top surface at which the incident wave is attenuated to 1/e of its initial value or when SE_A_ is higher than 9 dB (= 20 log (1/e)) [21, 22].

$\text{SE}_{\text{T}} = \text{SE}_{\text{R}} + \text{SE}_{\text{A}} + \text{SE}_{\text{MR}}$ Eq. 8

$\text{SE}_{\text{R}}\text{ = 10 log }\frac{\text{P}_{\text{I}}}{\text{P}_{\text{I}}\text{ - }\text{P}_{\text{R}}}\text{ = 10 log }\frac{\text{1}}{\text{1 - R}}\text{ = l0 log }\frac{\text{1}}{\text{1 - }\text{S}_{\text{11}}^{\text{2}}}\text{ }$ Eq. 9

$\text{SE}_{\text{R}}$= $\text{20 log }\frac{\text{Z}_{\text{0}}}{\text{4Z}_{\text{s}}}\text{= 39.5 + 10 log }\frac{\text{σ}}{\text{2πfμ}}$

$\text{SE}_{\text{A}}\text{ = }$10 log $\frac{\text{1 - R}}{\text{T}}$ = 10 log $\frac{\text{1 -}\text{ S}_{\text{11}}^{\text{2}}}{\text{S}_{\text{21}}^{\text{2}}}$ Eq. 10

$\text{SE}_{\text{A}}$ = $\text{20 log}$ $\text{e}^{\text{d/δ}}$ = 8.7 d $\sqrt{\text{σπfμ}}$

where the $\text{Z}_{\text{0}}$, $\text{Z}_{\text{s}}$, $\sigma$, $\text{f}$, $\mu$, 𝛿, and d represent the impedance of the air, the impedance of the shield, electrical conductivity, frequency, magnetic permeability, skin depth, and thickness of the sample. The impedance of the air is constant and equal to 377 Ω [16].

According to Eqs. 9 and 10, the absorption is a function of μ, σ, and d, while the reflection depends on σ/μ. That is why non-magnetic materials are prone to merely shield the EM waves by reflection. It is worth mentioning that the obligatory factor for using magnetic shields is the preservation of the material’s magnetic permeability at the intended frequency range. For instance, although ferromagnetic metals have a high permeability, their high conductivity provokes eddy current losses that cause the permeability to drop at lower frequencies than expected (50 KHz-10 MHz). In this regard, using the magnetic particles in a small size makes the effect of eddy current losses negligible. In superparamagnetic nanoparticles (like Fe_3_O_4_ nanoparticles used in this work) the magnetic spins fluctuate very quickly, and the relaxation is expected to occur at high frequencies (GHz) [22, 23].

# **References**

1. Darby, R., Chemical engineering fluid mechanics, revised and expanded. 2017: CRC Press.

2. Mahammedi, A., H. Ameur, and A. Ariss, Numerical investigation of the performance of kenics static mixers for the agitation of shear thinning fluids. Journal of Applied Fluid Mechanics, 2017. **10**(3): p. 989-999.

3. Bruel, C., et al., The structural amphiphilicity of cellulose nanocrystals characterized from their cohesion parameters. Carbohydrate polymers, 2019. **205**: p. 184-191.

4. Ho, M., et al., Direct Ink Writing of Conductive Hydrogels. Advanced Functional Materials, 2025: p. 2415507.

5. Hajian, A., et al., Understanding the dispersive action of nanocellulose for carbon nanomaterials. Nano letters, 2017. **17**(3): p. 1439-1447.

6. Shariatnia, S., et al., Hybrid cellulose nanocrystal-bonded carbon nanotubes/carbon fiber polymer composites for structural applications. ACS Applied Nano Materials, 2020. **3**(6): p. 5421-5436.

7. Li, Y., et al., Cellulose‐nanofiber‐enabled 3D printing of a carbon‐nanotube microfiber network. Small Methods, 2017. **1**(10): p. 1700222.

8. Liu, Y., et al., High-Performance Cellulose Nanofibers/Carbon Nanotubes Composite for Constructing Multifunctional Sensors and Wearable Electronics. Advanced Fiber Materials, 2024. **6**(3): p. 758-771.

9. Zakani, B., et al., Effect of particle concentration on lubrication performance of cellulose nanocrystalline (CNC) water-based lubricants: mixed lubrication regime. Cellulose, 2022. **29**(7): p. 3963-3984.

10. Li, M.C., et al., Rheological aspects of cellulose nanomaterials: Governing factors and emerging applications. Advanced Materials, 2021. **33**(21): p. 2006052.

11. Shafiei-Sabet, S., W. Hamad, and S. Hatzikiriakos, Ionic strength effects on the microstructure and shear rheology of cellulose nanocrystal suspensions. Cellulose, 2014. **21**: p. 3347-3359.

12. Yang, W., et al., Flexible and strong Fe3O4/cellulose composite film as magnetic and UV sensor. Applied Surface Science, 2020. **507**: p. 145092.

13. Hyun, K., et al., Large amplitude oscillatory shear as a way to classify the complex fluids. Journal of Non-Newtonian Fluid Mechanics, 2002. **107**(1-3): p. 51-65.

14. Kamkar, M., et al., Large amplitude oscillatory shear flow: Microstructural assessment of polymeric systems. Progress in Polymer Science, 2022. **132**: p. 101580.

15. Rau, D.A., M.J. Bortner, and C.B. Williams, A rheology roadmap for evaluating the printability of material extrusion inks. Additive Manufacturing, 2023. **75**: p. 103745.

16. Al-Saleh, M.H. and U. Sundararaj, Electromagnetic interference shielding mechanisms of CNT/polymer composites. Carbon, 2009. **47**(7): p. 1738-1746.

17. Neelakanta, P.S., Handbook of electromagnetic materials: monolithic and composite versions and their applications. 1995: CRC press.

18. Ghaffarkhah, A., et al., Functional Janus structured liquids and aerogels. Nature Communications, 2023. **14**(1): p. 7811.

19. Panahi‐Sarmad, M., et al., MOF‐Based Electromagnetic Shields Multiscale Design: Nanoscale Chemistry, Microscale Assembly, and Macroscale Manufacturing. Advanced Functional Materials, 2023: p. 2304473.

20. Paul, C.R., R.C. Scully, and M.A. Steffka, Introduction to electromagnetic compatibility. 2022: John Wiley & Sons.

21. Kaiser, K.L., Electromagnetic shielding. 2005: Crc Press.

22. Ribadeneyra, M.C., J.P. de Diego, and M.G. González, EMI shielding composites based on magnetic nanoparticles and nanocarbons. Unpublished Doctoral thesis, Universidad Carlos III de Madrid, 2014.

23. Habib, A., et al., The role of eddy currents and nanoparticle size on AC magnetic field–induced reflow in solder/magnetic nanocomposites. Journal of Applied Physics, 2012. **111**(7).
